# Factors governing the transcriptome changes and chronological lifespan of fission yeast during phosphate starvation

**DOI:** 10.1016/j.jbc.2024.105718

**Published:** 2024-02-02

**Authors:** Angad Garg, Ana M. Sanchez, Beate Schwer, Stewart Shuman

**Affiliations:** 1 Molecular Biology Program, Memorial Sloan Kettering Cancer Center, New York, New York, USA; 2 Gerstner Sloan Kettering Graduate School of Biomedical Sciences, New York, New York, USA; 3 Department of Microbiology and Immunology, Weill Cornell Medical College, New York, New York, USA

**Keywords:** phosphate starvation response, transcriptomics, transcription factor Pho7, ribosomal proteins, tRNA biogenesis, translation factors, aminoacyl tRNA synthetase

## Abstract

Starvation of *Schizosaccharomyces pombe* for inorganic phosphate elicits adaptive transcriptome changes in which mRNAs driving ribosome biogenesis, tRNA biogenesis, and translation are globally downregulated, while those for autophagy and phosphate mobilization are upregulated. Here, we interrogated three components of the starvation response: upregulated autophagy; the role of transcription factor Pho7 (an activator of the *PHO* regulon); and upregulated expression of *ecl3*, one of three paralogous genes (*ecl1*, *ecl2*, and *ecl3*) collectively implicated in cell survival during other nutrient stresses. Ablation of autophagy factor Atg1 resulted in early demise of phosphate-starved fission yeast, as did ablation of Pho7. Transcriptome profiling of phosphate-starved *pho7*Δ cells highlighted Pho7 as an activator of genes involved in phosphate acquisition and mobilization, not limited to the original three-gene *PHO* regulon, and additional starvation-induced genes (including *ecl3*) not connected to phosphate dynamics. Pho7-dependent gene induction during phosphate starvation tracked with the presence of Pho7 DNA-binding elements in the gene promoter regions. Fewer ribosome protein genes were downregulated in phosphate-starved *pho7*Δ cells *versus* WT, which might contribute to their shortened lifespan. An *ecl3*Δ mutant elicited no gene expression changes in phosphate-replete cells and had no impact on survival during phosphate starvation. By contrast, pan-*ecl* deletion (*ecl123*Δ) curtailed lifespan during chronic phosphate starvation. Phosphate-starved *ecl123*Δ cells experienced a more widespread downregulation of mRNAs encoding aminoacyl tRNA synthetases vis-à-vis WT or *pho7*Δ cells. Collectively, these results enhance our understanding of fission yeast phosphate homeostasis and survival during nutrient deprivation.

Inorganic phosphate is an essential nutrient acquired by cells from their environment. Cells from all domains of life respond to acute phosphate starvation by inducing the transcription of phosphate acquisition genes. In the fission yeast *Schizosaccharomyces pombe*, phosphate acquisition (*PHO*) genes *pho1* (cell surface acid phosphatase), *pho84* (phosphate transporter), and *tgp1* (glycerophosphodiester transporter) are repressed under phosphate-replete conditions by upstream long noncoding RNA (lncRNA)-mediated transcriptional interference (1) and derepressed during acute phosphate starvation over 4 h (2). Induction of the *PHO* regulon during acute phosphate starvation depends on the transcription factor Pho7 that binds to target DNA sequences in the *PHO* mRNA promoters (3, 4, 5, 6).

In a recent study, we characterized the adaptations of fission yeast to chronic phosphate starvation, during which cells enter a state of quiescence, initially fully reversible upon replenishing phosphate after 2 days of starvation (7). Time-resolved analyses of transcriptome changes revealed coherent perturbations of gene expression, whereby the mRNAs encoding the cellular machineries for ribosome biogenesis, tRNA biogenesis, and protein translation are globally downregulated, whereas those for autophagy and phosphate mobilization are upregulated (Fig. S1). At the proteome level, phosphate starvation resulted in depletion of ribosome assembly factors, 60S and 40S proteins, tRNA-modifying enzymes, and translation factors. The phosphate starvation-induced downregulation of ribosome biogenesis genes, ribosomal protein genes, translation factor genes, and tRNA biogenesis genes was abolished or severely curtailed in the presence of cycloheximide (7). Also, the upregulation of the *PHO* genes *pho84*, *pho1*, and *tgp1* was squelched by cycloheximide treatment due to a failure to shut off the production of upstream interfering lncRNAs. We surmised that phosphate starvation-induced transcriptional shut-off of the translation machinery mRNAs and *PHO*-regulatory lncRNAs requires new synthesis of one or more repressive proteins.

WT fission yeast cells progressively lose viability in the interval between 2 days (100% viable), 14 days (44% viable), and 28 days (4% viable) of phosphate starvation (7). We hypothesized that prolonged phosphate deficiency sets up bifurcated pathways of cell quiescence or cell death and that the balance between these outcomes might be altered by mutations in genes that affect these pathways. An initial clue to pathway choice was that *maf1* mRNA and Maf1 protein were upregulated during a 48-h period of phosphate starvation. Maf1 is an endogenous negative regulator of RNA polymerase III (Pol3) transcription. We proceeded to show that *maf1*Δ fission yeast cells undergo accelerated demise between 1 and 2 days of phosphate starvation. Starved *maf1*Δ cells upregulate genes for phosphate mobilization and autophagy and downregulate the machinery for production and processing of rRNA and tRNA and for protein synthesis akin to starved WT cells. The 24 to 48 h temporal window during which phosphate-starved *maf1*Δ cells begin to expire is associated with overproduction of tRNA and the accumulation of polyadenylated tRNAs, intron-containing pre-tRNAs, and unspliced tRNA fragments (7). We proposed that Maf1 prolongs chronological lifespan during phosphate starvation by repressing Pol3 and preventing a death pathway associated with aberrant tRNA metabolism.

In the present study, we focus on three different arms of the transcriptional response to chronic phosphate starvation: (i) upregulated autophagy; (ii) Pho7-dependent transcription; and (iii) upregulated expression of *ecl3*, one of three paralogous genes (*ecl1*, *ecl2*, and *ecl3*) that are collectively implicated in cell survival during nutrient stress.

## Results

### Limited transcriptome changes upon transition of fission yeast from YES medium to phosphate-replete ePMGT medium

Our phosphate starvation protocol entailed transfer of log-phase cultures of *S. pombe* cells grown at 30 °C from YES medium (yeast extract with supplements) to a defined medium ePMGT (enhanced Pombe Minimal Glutamate with Thiamine) containing either 15.5 mM phosphate (ePMGT+PO_4_) or lacking phosphate (ePMGT–PO_4_). Fission yeast continued to grow normally after transfer from YES to ePMGT+PO_4_, that is, the doubling time of 147 min in ePMGT+PO_4_ was virtually identical to the doubling time of 143 min in YES (7), and there was no increase in cell surface acid phosphatase activity (a sensitive indicator of phosphate starvation response). Upon transfer from YES to ePMGT–PO_4_ (starvation medium), cells rapidly accumulated surface acid phosphatase (Pho1) and underwent two to three rounds of cell division before entering a state of G0 quiescence, as gauged by flow cytometry. Previously, we interrogated the transcriptional adaptations to phosphate starvation by performing RNA-seq on poly(A)^+^ RNA isolated from WT fission yeast cells prior to (time 0) and 4, 8, 12, 24, 36, and 48 h after transfer from YES to ePMGT–PO_4_. This analysis revealed that the mRNAs encoding the cellular machineries for ribosome biogenesis, tRNA biogenesis, and protein translation are globally downregulated, whereas those for autophagy and phosphate mobilization are upregulated (7). Transfer from YES to ePMGT–PO_4_ also resulted in upregulation at 4 h of a subset of core environmental stress response genes that had been identified by Bähler and colleagues (8) as induced in response to oxidation, heavy metal exposure, heat shock, osmotic stress, and DNA damage, thereby implying that fission yeast cells experience acute phosphate starvation as a stress.

Although we attributed the observed transcriptome changes *versus* the YES time 0 dataset solely to phosphate limitation, we proceeded here to query whether and how the change from complex to defined phosphate-replete medium might impact gene expression, by performing RNA-seq on poly(A)^+^ RNA isolated from WT fission yeast cells prior to (time 0) and 2, 4, 8 h after transfer from YES to ePMGT+PO_4_. Three biological replicates were sequenced for each time point. As before, we imposed the criterion that genes be differentially expressed ±2-fold at two or more of the time points to be deemed genuinely responsive to the switch in media. We thereby identified 72 annotated protein-coding transcripts that were upregulated by between 2-fold and 90-fold in ePMGT+PO_4_ (Table S1). Constituents of the fission yeast iron homeostasis regulon (n = 9) and noniron transmembrane transporters (n = 21) were the predominant class of genes upregulated during the change to ePMGT+PO_4_. Forty seven of the 72 genes up in ePMGT+PO_4_ were also upregulated after transfer from YES to ePMGT–PO_4_. We attribute the increased expression of iron regulon genes to the fact that the concentration of iron in YES (3.2 μM) is 4-fold higher than in ePMGT (0.74 μM).

The key point here is that growth in ePMGT+PO_4_ did not increase the expression of the key genes highlighted previously that were induced at equivalent times during phosphate starvation in ePMGT–PO_4_, that is, 25 autophagy genes, 23 Pol2 transcription factors, the Pol3 repressor Maf1, and 40 phosphate-mobilizing proteins/enzymes (7). Two transcription factors (Cbf12, Toe1) that were noted previously to be upregulated in ePMGT–PO_4_ (7) were upregulated to a similar extent in ePMGT+PO_4_. Two phosphate-mobilizing genes—*SPBPB2B2.06c* and *SPAC1039.02*—encoding predicted extracellular 5′-nucleotidase enzymes (which hydrolyze extracellular AMP to adenosine and phosphate) that were upregulated by 21,000-fold and 140-fold, respectively, after 8 h in ePMGT–PO_4_ (7) were increased to a lesser extent after 8 h in ePMGT+PO_4_ (by 13-fold and 4-fold, respectively) from which we conclude that they are *bona fide* phosphate-regulated genes.

Only five coding genes were downregulated by 2- to 4-fold after transfer from YES to ePMGT+PO_4_. Here, the salient point is that growth in ePMGT+PO_4_ did not result in downregulation of the several hundred ribosome biogenesis, tRNA biogenesis, and protein translation genes seen during an equivalent interval of phosphate starvation (7).

### Autophagy prolongs the lifespan of phosphate-starved fission yeast

Autophagy is an inducible response of eukaryal cells to changes in their environment, such as nutrient deprivation, whereby cytoplasmic components are enclosed within membranous autophagosomes that undergo lysosome/vacuole fusion, leading to degradation of the autophagosome contents (9). Autophagy thereby allows for salvage of the building blocks of preexisting macromolecules to restore nutrients. Autophagy is initiated and executed by a cascade of ubiquitin-like conjugation and kinase events performed by a conserved set of “Atg” proteins, named after their budding yeast orthologs (9), in concert with additional autophagy factors, none of which is essential for vegetative fission yeast growth under nutrient-rich conditions (10). A key finding from our RNA-seq experiments was that 25 of the fission yeast genes in the autophagy pathway were upregulated at the transcriptional level in response to phosphate starvation (Fig. S1). The greatest fold increases were noted for mRNAs specifying Atg12 (a ubiquitin-like protein modifier conjugated to Atg5) and Atg1 (a serine/threonine protein kinase), which were up by 10-fold and 5-fold, respectively. Proteomics analysis showed that the levels of 12 of the autophagy proteins were increased by 2- to 12-fold within 24 h of phosphate starvation, including Atg1, which increased ∼4-fold (7).

**Figure 1:**
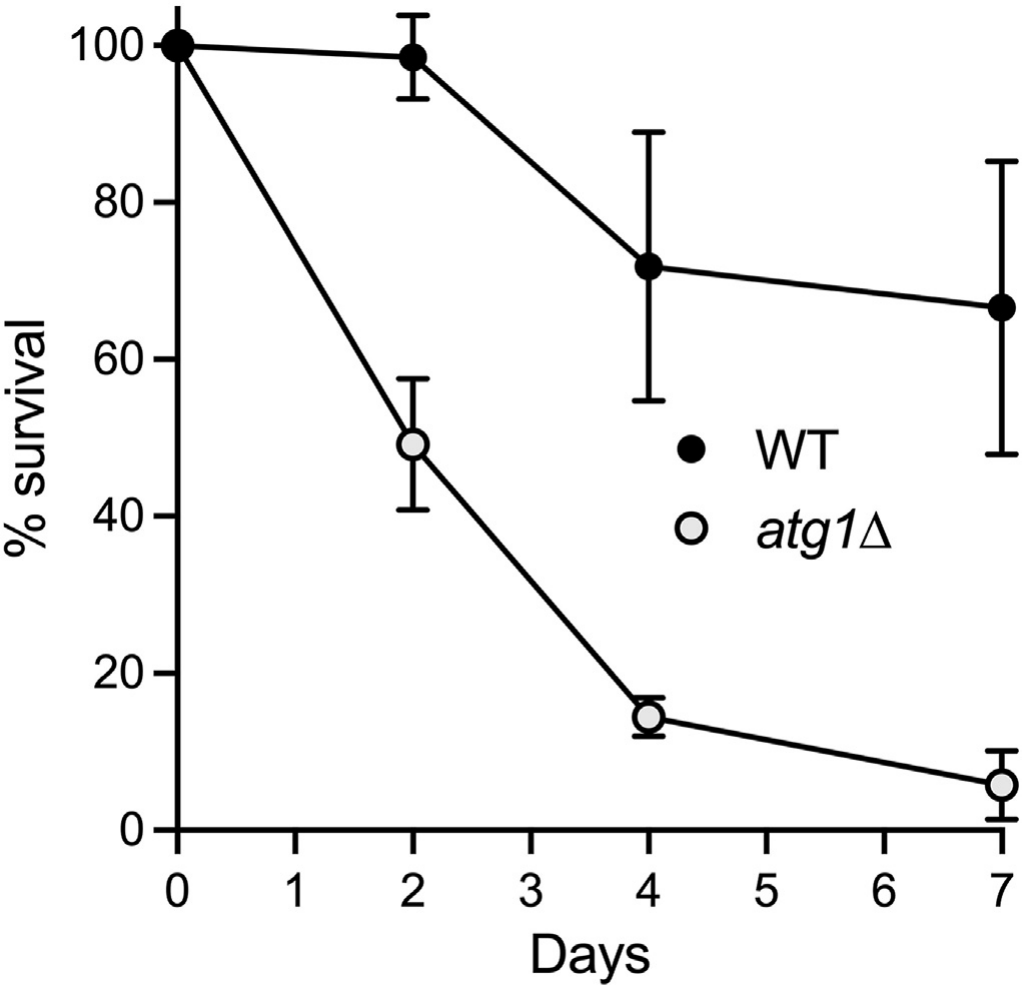
**Deletion of *atg1* shortens survival during phosphate starvation.** Viable colony counts of phosphate-starved WT and *atg1*Δ cultures were normalized to the time 0 control (100%). Percent survival is plotted as a function of starvation time. The 2-day survival data are the average of four independent experiments ± SD. The 4-day survival data are the average of five experiments ± SD. The 7-day WT survival data are the average of eight experiments ± SD. The 7-day *atg1*Δ survival data are the average of three experiments ± SD.

A pertinent question is whether the transcriptional induction and increased production of autophagy pathway proteins aid in prolonging the chronological lifespan of phosphate-starved fission yeast once they become quiescent. To address this point, we deleted the *atg1* gene and compared the survival of WT and *atg1*Δ cells that were subjected to phosphate starvation then allowed to recover growth on phosphate-replete medium. Aliquots of cells were collected prior to (time 0) and 2, 4, and 7 days after transfer from YES to ePMGT–PO_4_ and counted with a hemacytometer. Serial dilutions were plated on YES agar medium and incubated at 30 °C. Viable colony counts were normalized to the time 0 control and percent survival was plotted as a function of starvation time (Fig. 1). WT cells retained full viability after 2 days of starvation before gradual demise ensued (66% survival after 7 days of starvation). The instructive finding was that deletion of Atg1 resulted in accelerated death of phosphate-starved cells, with 50% survival after 2 days of phosphate deprivation and less than 6% survival after 7 days (Fig. 1).

### Interrogating the role of transcription factor Pho7

The 738-amino acid Pho7 protein is a member of the zinc binuclear cluster (Zn_2_-Cys_6_) family of fungal DNA-binding transcription regulators (11). Dennis Wykoff and colleagues identified Pho7 as a key player in phosphate homeostasis by screening a fission yeast gene KO collection for strains defective in the elaboration of Pho1 cell surface acid phosphatase activity in response to phosphate starvation (3). They showed that *pho7* deletion interdicted the phosphate starvation-induced increase in mRNAs encoding Pho1 acid phosphatase, the Pho84 phosphate transporter, and the Tgp1 glycerophosphodiester transporter (2, 3). These three genes comprise a *PHO* regulon; their expression is repressed under phosphate-replete conditions by lncRNA-mediated transcriptional interference (1) and derepressed during phosphate starvation when lncRNA synthesis abates (7). Microarray analyses of gene expression in *pho7*^+^ and *pho7*Δ strains in phosphate-replete and phosphate-starved cells affirmed the role of Pho7 in the acute phosphate starvation response, while highlighting an additional function of Pho7 in driving expression of multiple stress-response genes independent of phosphate status (2). Their microarray analysis identified 149 protein-coding genes that were upregulated at least 2-fold at 4 h of phosphate starvation, 113 of which were upregulated by at least 2-fold at 4 h in our RNA-seq analysis of phosphate starvation. Carter-O’Connell *et al*. (2) noted that one-third of the set of 22 genes induced after 2 h of phosphate starvation were *pho7*-dependent. In keeping with the greater sensitivity of RNA-seq, we detected 710 mRNAs that were upregulated at 4 h and at least one other time point (7). Thus, we were interested in applying RNA-seq to a *pho7*Δ strain to more fully delineate a “Pho7 regulon” and its impact on adaptation to phosphate starvation.

### Transcriptional profiling of *pho7Δ* cells under phosphate-replete conditions

**Figure 2:**
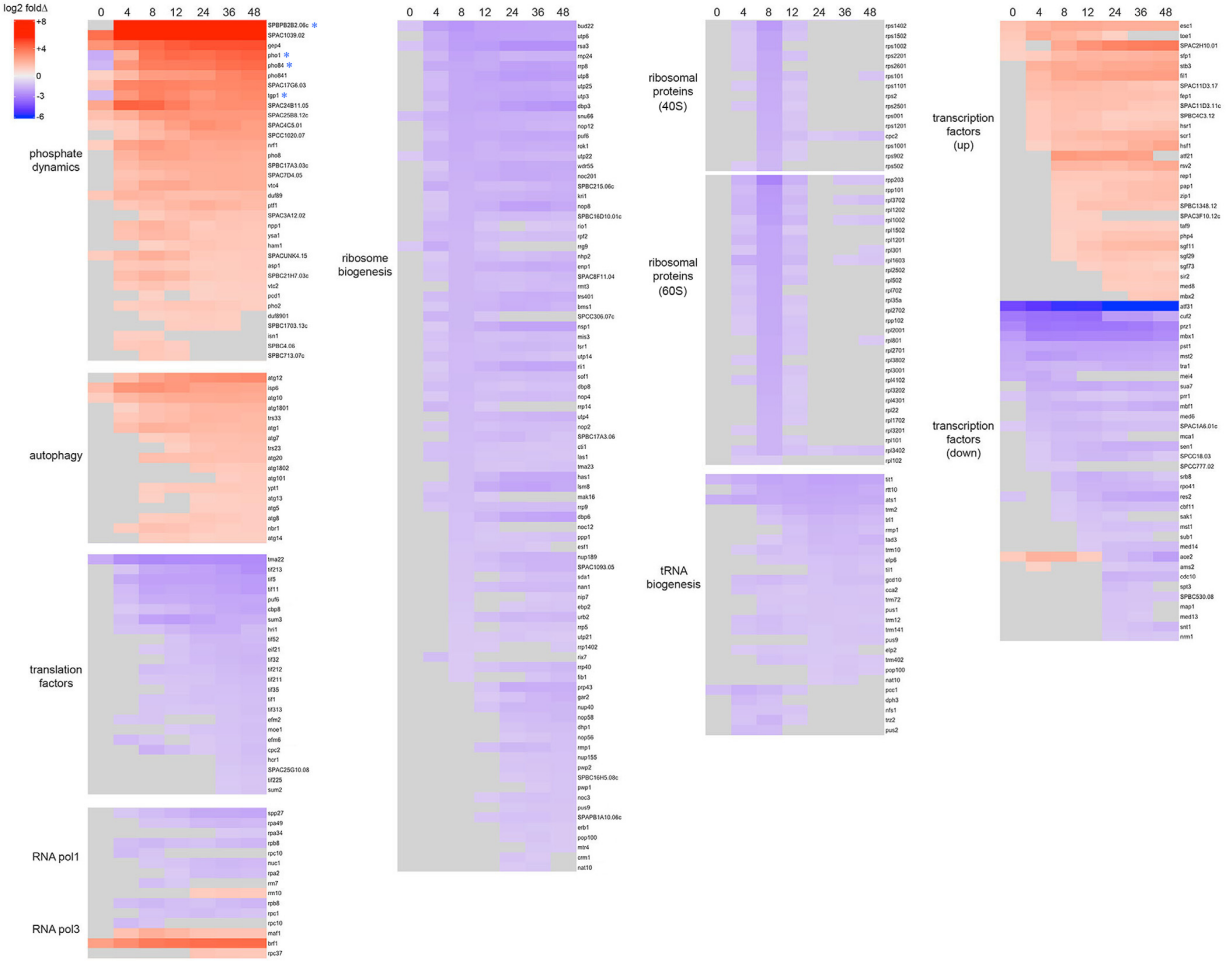
**Transcriptome changes in *pho7*Δ cells during phosphate starvation.** poly(A)^+^ RNA-seq data from *pho7*Δ cells grown in phosphate-replete conditions (0 h) or after 4, 8, 12, 24, 36, and 48 h of phosphate starvation was compared to that of WT cells grown in phosphate-replete conditions. The log2 fold changes of individual mRNAs, output in DESeq2, within the indicated functional classes of genes are colored according to the scale shown on the *top left*. *Red* signifies upregulation and *blue* signifies downregulation. *Gray shading* denotes less than a 2-fold change (and/or *p* value >0.05) in transcript level at the times specified. Each row represents a gene (specified at *right*), and each column represents the indicated sampling time (h). Pho7-dependent genes are denoted by *blue asterisks*.

We performed RNA-seq on poly(A)^+^ RNA isolated from WT and *pho7*Δ cells during logarithmic growth in YES medium at 30 °C. Three biological replicates were sequenced for each strain. A cut-off of ±2-fold change in normalized transcript read level in *pho7*Δ *versus* WT and a *p* value of ≤0.05 were the criteria applied to derive a list of differentially expressed genes (Table S2). We thereby identified 361 annotated protein-coding RNAs that were downregulated in phosphate-replete *pho7*Δ cells (by between 2- and 86-fold), including those of known *PHO* regulon genes *pho1*, *pho84*, and *tgp1* (each down by ∼3-fold) and *pho842* and *pho843* encoding inorganic phosphate transmembrane transporters (down by 24-fold and 2-fold, respectively). The other *pho7*Δ downregulated mRNAs (15 of which were decreased by more than 16-fold) had no obvious connections to phosphate homeostasis. mRNAs encoding several DNA-binding transcription factors were downregulated in *pho7*Δ cells: Atf31 (down 24-fold); Cuf2 (9-fold), Prz1 (7-fold), and Mbx1 (5-fold) (Fig. 2, time 0).

We were surprised to find that 385 protein-coding RNAs were upregulated in phosphate-replete *pho7*Δ cells (by between 2- and 72-fold). Of the genes identified presently as upregulated in phosphate-replete *pho7*Δ cells by RNA-seq, only four—*SPAC1039.02* (up 17-fold), *urg2* (up 16-fold), *urg1* (up 14-fold), and *SPAC521.03* (up 8-fold)—had been flagged as upregulated in the earlier microarray analyses of phosphate-replete *pho7*Δ cells by Carter-O’Connell *et al*. (2). It is noteworthy that our RNA-seq analysis showed that these same four genes were upregulated during the shift of WT cells from YES to ePMGT+PO_4_ as follows: *SPAC1039.02* (up 8-fold), *urg2* (up 20-fold), *urg1* (up 32-fold), and *SPAC521.03* (up 4-fold). Indeed, a total of 30 of the mRNAs that were upregulated in phosphate-replete *pho7*Δ cells were also upregulated during the shift of WT cells from YES to ePMGT+PO_4_ (Table S1). This suggests that absence of Pho7 might simulate a stress-like state in cells growing in rich YES medium. This is consistent with the fact that the *pho7*Δ strain displays severe *ts* and *cs* growth defects on YES agar at 37 °C and 18 °C, respectively (4).

### *pho7*Δ cells mount a global transcriptional response to phosphate starvation

We performed RNA-seq on poly(A)^+^ RNA isolated from *pho7*Δ cells harvested 4, 8, 12, 24, 36, and 48 h after transfer from YES to ePMGT–PO_4_ medium. Three biological replicates were sequenced for each time point. A cut-off of ±2-fold change in normalized transcript read level compared to the WT time 0 control read level (*i.e.*, in YES medium prior to starvation) and a *p* value of ≤0.05 were the criteria applied to derive an initial list of differentially expressed genes as a function of starvation time. A secondary criterion was that genes be differentially expressed at two or more time points to be deemed genuinely responsive to phosphate starvation. We mined the RNA-seq data to see whether loss of Pho7 interdicted any of the adaptive and (presumably) lifespan-prolonging arms of the transcriptional reprogramming observed in starved WT cells (7). We found that *pho7*Δ cells manifested a *forme fruste* of the WT gene expression pattern (shown as a heat map in Fig. S1) in which genes involved in phosphate mobilization and autophagy were upregulated and the protein synthetic machinery was downregulated in response to phosphate starvation, albeit with fewer ribosome subunit genes affected in *pho7*Δ cells *versus* WT (heat map in Fig. 2). To summarize: (i) mRNAs encoding 34 proteins involved in phosphate dynamics were upregulated; (ii) mRNAs encoding 17 proteins driving autophagy were upregulated; (iii) mRNAs for 29 proteins of the 60S ribosome and 15 proteins of the 40S ribosome were transiently downregulated at 4 to 12 h (*versus* 129 ribosomal protein mRNAs that were downregulated in WT cells; Fig. S1); (iv) eight protein components of the Pol1 transcription machinery were downregulated; (v) 85 ribosome assembly factors were downregulated (*versus* 115 ribosome assembly factors downregulated in WT cells; Fig. S1); (vi) three constituents of the Pol3 transcription machinery were downregulated, while the mRNAs encoding Rpc37, TFIIIB subunit Brf1, and the Pol3 repressor Maf1 were upregulated; (vii) 26 tRNA biogenesis enzymes were downregulated; and (viii) 24 translation factors were downregulated (Table S3 and Fig. 2).

**Figure 3:**
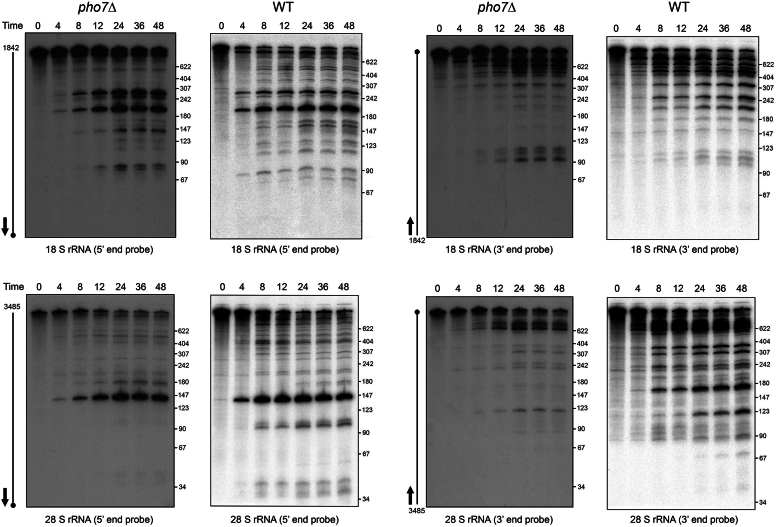
**Phosphate starvation of *pho7*Δ cells triggers endonucleolytic cleavage of 18S and 28S rRNA**. Total RNAs (5 μg) from *pho7*Δ cells harvested prior to (0 h) and 4, 8, 12, 24, 36, and 48 h after transfer to phosphate-free medium were resolved by urea-PAGE in parallel with 5′ ^32^P-labeled denatured DNA size markers (pBR322 MspI digest ladder). The gel contents were electro-transferred to a nylon membrane, which was then serially hybridized to ^32^P-labeled DNA oligonucleotide probes complementary to the 5′ and 3′ ends of 18S rRNA (*top panels*) and 28S rRNA (*bottom panels*) as described previously (7). Annealed probes were detected by autoradiography. The positions and sizes (nt) of the DNA markers are indicated on the *right*. The 18S and 28S rRNAs are depicted alongside the gels as *thin lines with dots* indicating the 5′ ends. The annealed 5′ end and 3′ end oligonucleotide probes are shown as *thick lines with arrows* indicating the 3′ end of the probe. The *pho7*Δ Northern blots are displayed adjacent to analogous Northern blots for 18S and 28S rRNAs from phosphate-starved WT cells reported previously (7).

We showed previously that phosphate starvation of WT cells elicits endonucleolytic cleavages of 18S and 28S rRNAs to generate specific fragments that accumulated by 8 h and persisted for 48 h of starvation (7; data reprised in Fig. 3). The apparent site-specificity of the rRNA cleavages and the stability of the cleavage products suggested that the starvation-induced repression of ribosomal protein mRNAs and accompanying depletion of ribosome proteins exposes regions of the rRNAs to cellular endonucleases. The bioanalyzer profiles of the total RNA samples from three biological replicates of *pho7*Δ cells indicated that the large ribosomal RNAs underwent fragmentation during the starvation time course. In the new experiments presented in Figure 3, we tracked the fate of 18S and 28S rRNA during phosphate starvation of *pho7*Δ cells *via* Northern blotting with ^32^P-labeled oligonucleotide probes complementary to the very 5′ and 3′ ends of the mature 1842-nt 18S rRNA and 3485-nt 28S rRNA. We observed a starvation time-dependent accumulation of stable rRNA fragments cleaved at discrete internal sites at distance from the 5′ and 3′ ends corresponding to their size. The major cleavage products detected in starved *pho7*Δ cells with the 5′ probes were the same as those detected in WT cells (Fig. 3). Whereas the larger (>400 nt) rRNA fragments detected with the 3′ probes were the same as in WT cells, 3′ fragments smaller than 400 nt were less abundant in *pho7*Δ cells vis-à-vis WT (Fig. 3), likely because fewer ribosomal protein mRNAs were downregulated by phosphate starvation in *pho7*Δ cells.

### Pho7-dependent gene induction during long-term phosphate starvation

**Figure 4:**
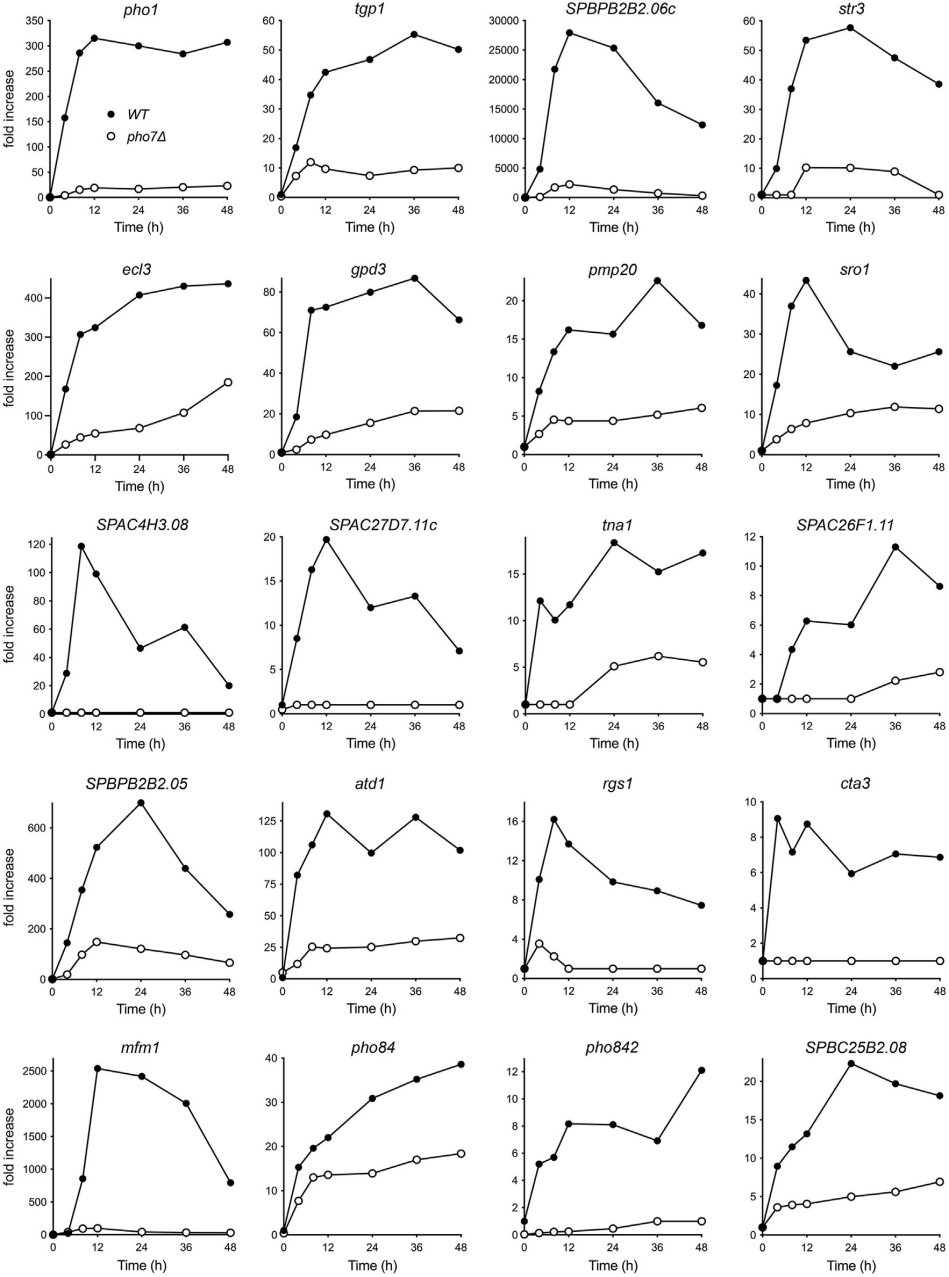
**Pho7-dependent gene induction during long-term phosphate starvation.** The fold increases in the indicated mRNA levels in phosphate-starved WT (•) *versus pho7*Δ (○) cells, normalized to the time 0 control are plotted as a function of starvation time. Fold increase is plotted on a linear scale on the *y*-axis; the values are derived from the log2 fold increases determined from RNA-seq experiments and output in DESeq2. The log2 fold change SE values ranged from 0.052 to 0.67 (corresponding to linear fold change SE values of 1–1.6) and are not visible on the graphs because they are smaller than the data symbols. SE, standard error.

Focusing on the genes that were upregulated during phosphate starvation of WT cells, we set a 4-fold decrement in mRNA induction at two or more times during phosphate starvation of *pho7*Δ cells as the criterion for designating a starvation-induced gene as Pho7-dependent. The expression profiles of 19 such genes are shown in Figure 4. The dependencies on Pho7 were high for several genes involved in phosphate mobilization. To wit: (i) *SPBPB2B2.06c* encoding an extracellular 5′-nucleotidase is the most highly upregulated mRNA in phosphate-starved WT cells in terms of fold induction, by a factor of 28,500 at 12 h, but is only up by 2200-fold in *pho7*Δ cells; (ii) *pho1* mRNA increased by 315-fold at 12 h in WT cells compared with 19-fold in *pho7*Δ cells; (iii) *tgp1* mRNA was upregulated by 55-fold at 36 h in WT cells *versus* 9-fold in *pho7*Δ cells; and (iv) *pho842* expression at 12 h in WT cells was 32-fold higher than in *pho7*Δ cells. Of the three canonical *PHO* regulon genes, *pho84* (upregulated by 39-fold in WT cells) was the least dependent on Pho7 for its expression during phosphate starvation, which was reduced by only a factor of 2 in *pho7*Δ versus WT (Fig. 4).

Other genes, seemingly unconnected to phosphate dynamics, were also strongly induced by phosphate starvation (by 9- to 2500-fold) in a manner dependent on Pho7 (Fig. 4). These genes encode proteins with known or predicted functions as follows: *str3* (heme transporter); *ecl3* (putative extender of chronological lifespan); *gpd3* (glyceraldehyde-3-phosphate dehydrogenase); *pmp20* (thioredoxin-related chaperone); *sro1* (stress-responsive “orphan” protein); *SPAC4H3.08* (3-hydroxyacyl-CoA dehydrogenase); *SPAC27D7.11c* (cell surface protein); *tna1* (carboxylic acid transmembrane transporter); *SPBPB2B2.05* (class I glutamine amidotransferase); *atd1* (aldehyde dehydrogenase); *rgs1* (regulator of G-protein signaling); *cta3* (calcium transporting P-type ATPase); *mfm1* (M-factor mating pheromone precursor); *SPBC25B2.08* (pombe-specific protein); and *SPAC26F1.11* (unknown). Five of these genes—*str3*, *rgs1*, *mfm1*, *SPBPB2B2.05*, *SPAC27D7.11c*, and *SPAC4H3.08*—had been flagged as Pho7-dependent *via* microarray analyses of WT and *pho7*Δ cells under a variety of stress conditions (2).

### Pho7-dependent induced genes with Pho7-binding sites in their promoters comprise a Pho7 regulon

The RNA-seq data *per se* do not discriminate whether the contributions of Pho7 to the expression of specific genes are direct or indirect. Indirect effects apply if deleting *pho7* affects the transcription of genes that regulate the expression of other genes identified as Pho7-dependent, whereas direct regulation by Pho7 implies that Pho7 binds to one or more *cis*-acting DNA sequence elements in the promoter of the Pho7-dependent gene. The Pho7 DNA-binding domain (DBD) comprises the polypeptide segment from aa 279 to 368 that includes the Zn_2_Cys_6_ module. DNase footprinting and electrophoretic mobility shift assays located the Pho7 DBD recognition sites in the promoters of the *pho1* and *tgp1* genes to a 12-nt motif 5′-TCG(G/C)(A/T)xxTTxAA (4). The *pho1* promoter contains two Pho7 12-mer recognition elements (sites 1 and 2) in direct repeat orientation and separated by a 20-nt spacer. The Pho7-DBD binds independently and noncooperatively to these two sites in the *pho1* promoter. By contrast, the *tgp1* promoter contains a single Pho7 binding site. Crystal structures of Pho7 DBD in complex with its target site in the *tgp1* promoter (5′-TCGGACATTCAAAT) or site 2 in the *pho1* promoter (5′-TCGGAAATTAAAAA) highlighted two distinctive features of the Pho7 DBD: (i) it binds DNA as a monomer, unlike most other fungal zinc-cluster factors that bind as homodimers and (ii) it makes extensive interactions with its asymmetric target sequence over a 14-bp footprint that entails direct and/or water-mediated hydrogen bonding to individual nucleobases and backbone phosphates within, and remote from, the CGG triplet typically recognized by other Zn_2_Cys_6_ DBDs (5, 6). Comparison of the two Pho7 DBD–DNA structures revealed shared determinants of target site specificity as well as variations in the protein-DNA interface that accommodate different promoter DNA sequences. Nucleobase mutations in either of the *pho1* sites or in the *tgp1* site that eliminate Pho7 DBD binding *in vitro* result in loss of *pho1* or *tgp1* promoter activity *in vivo*, to the same extent as does deleting the *pho7* gene (4).

Using epitope-tagged Pho7 (Pho7-TAP), Carter O’Connell *et al*. (2) performed chromatin immunoprecipitation-seq analyses that identified 1676 peaks of Pho7 occupancy in the genome of phosphate-replete fission yeast cells. They found that the occupancy of 367 sites increased during phosphate starvation. Whereas it was not possible at the time to discern a Pho7 DNA binding motif, a revisited *in silico* analysis of the separate phosphate-starved and phosphate-replete Pho7-TAP chromatin immunoprecipitation-seq datasets identified a consensus dodecamer, 5′-TCG(G/C)AxTTTxAA, that agreed with the Pho7 DBD sites in the *pho1* and *tgp1* promoters. The prevalence of genomic Pho7 sites *in vivo*, and the suggestion that their occupancy is dynamic with phosphate status, accords with the present RNA-seq data on the number of genes for which expression is altered when Pho7 is absent.

To query which Pho7-dependent protein-coding genes shown in Figure 4, in addition to *pho1*, *tgp1*, and *pho84*, are likely to be directly regulated by Pho7, we inspected the genomic sequences upstream of the mapped transcription start sites (12) for Pho7 binding motifs containing the 5′-TCG(G/C) element or its inverse 5′-(C/G)CGA that is directly recognized by the Pho7 DBD. The transcription start sites are preceded by a TATA-box, upstream of which is at least one, and as many as five, candidate Pho7 DNA-binding sites, in either forward or inverted orientation (Fig. S2). We propose that this gene set comprises a phosphate starvation-responsive Pho7 regulon.

### Pho7 contributes to the chronological lifespan of phosphate-starved fission yeast

**Figure 5:**
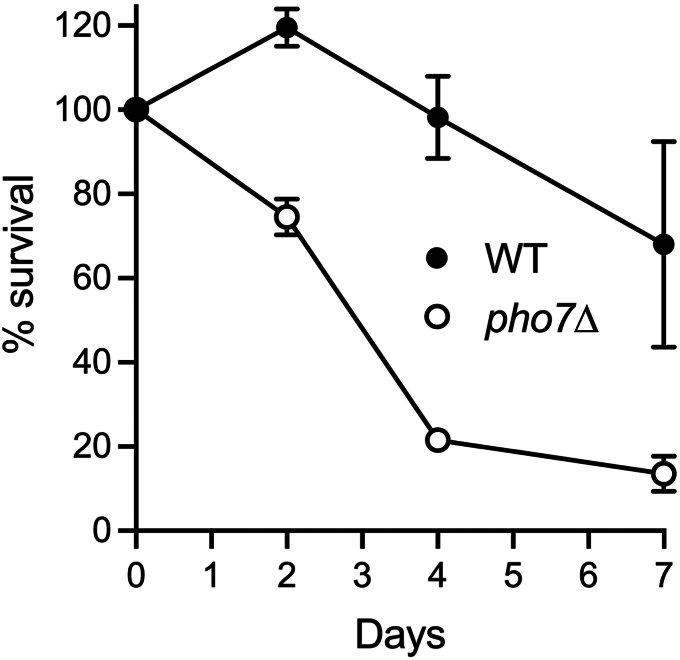
**Deletion of *pho7* shortens survival during phosphate starvation.** Viable colony counts of phosphate-starved WT and *pho7*Δ cultures were normalized to the time 0 control (100%). Percent survival is plotted as a function of starvation time. Each datum in the graph is the average of three independent experiments ± SD.

We maintained quiescent WT and *pho7*Δ cells at 30 °C in phosphate starvation ePMGT–PO_4_ medium and gauged their survival upon restoration of growth on phosphate-rich YES medium. *pho7*Δ cells displayed reduced viability after 4 and 7 days of starvation (22% and 14% survival, respectively) (Fig. 5). We surmise that Pho7 promotes the expression of gene(s) that extend the chronological lifespan of phosphate-starved cells.

### *ecl3*, alone among the three-member *ecl* gene family, is upregulated by phosphate starvation

**Figure 6:**
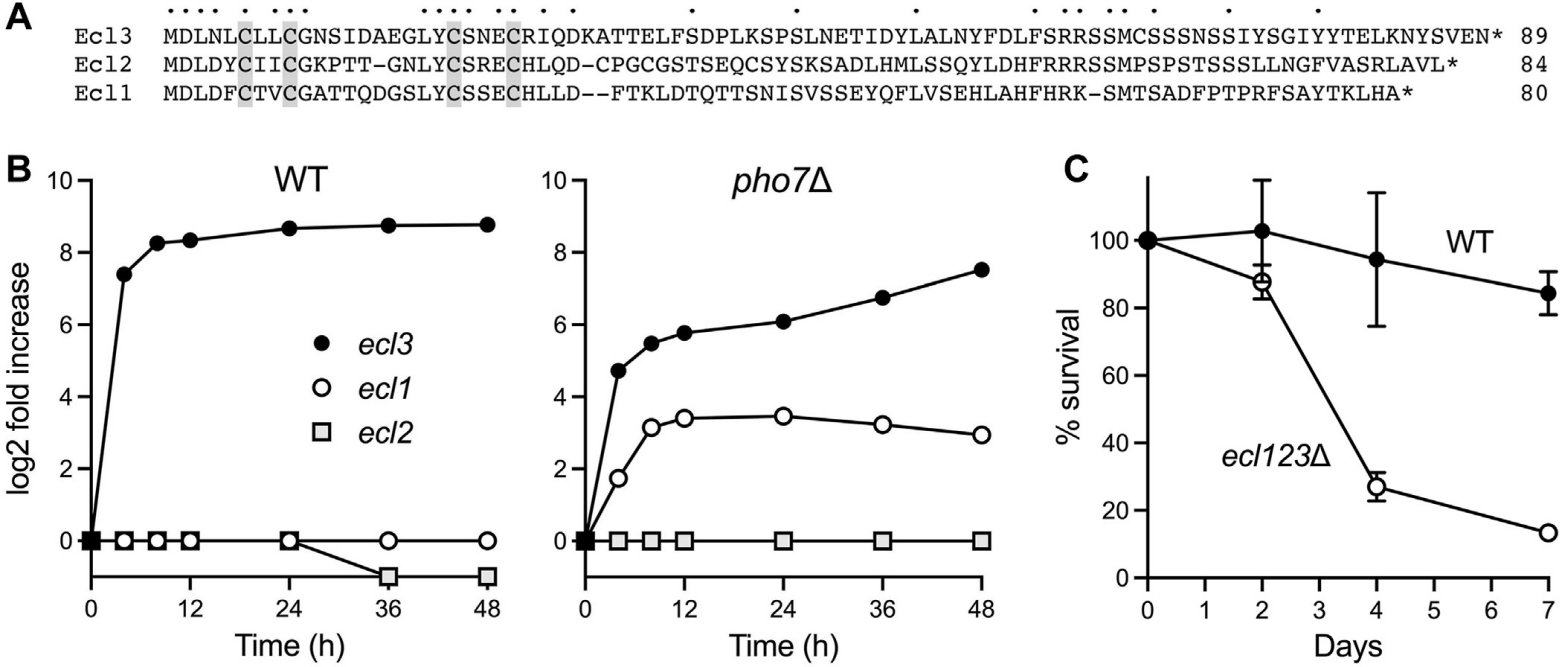
**Expression and function of the *ecl* gene family during phosphate starvation.***A*, alignment of the primary structures of the fission yeast Ecl1, Ecl2, and Ecl3 polypeptides. Positions of amino acid side chain identity/similarity are denoted by *dots* above the alignment. The putative zinc-binding cysteines are shaded in *gray*. *B*, the log2 changes in *ecl1*, *ecl2*, and *ecl3* mRNA levels in phosphate-starved WT (*left panel*) and *pho7*Δ (*right panel*) cells *versus* time 0 controls are plotted as a function of starvation time. *C*, pan-*ecl* deletion shortens survival during phosphate starvation. Viable colony counts of phosphate-starved WT and *ecl123*Δ cultures were normalized to the time 0 control (100%). Percent survival is plotted as a function of starvation time. The 2-day survival data are the average of five independent experiments ± SD. The 4-day survival data are the average of four independent experiments ± SD. The 7-day survival data are the average of three independent experiments ± SD.

The fission yeast polypeptides Ecl1 (80 aa), Ecl2 (84 aa), and Ecl3 (89 aa) comprise a family of protein paralogs containing an N-terminal tetracysteine zinc-binding motif—**C**xx**C**G….LY**C**SxE**C** (13, 14) (Fig. 6*A*). The name Ecl (extender of chronological lifespan) derives from initial findings that the *ecl1*, *ecl2*, or *ecl3* genes on a high-copy plasmid extended the viability of WT cells after entry into stationary phase (13, 15). Subsequent studies by the Aiba lab showed that the expression of the three *ecl* genes is responsive to distinct signals, as follows: (i) *ecl1* mRNA increased by ∼100-fold in sulfate-starved cells in a manner that depends on transcription factor Zip1 (16); (ii) *ecl2* expression was increased only 3-fold by sulfate starvation, whereas *ecl3* expression was unchanged (16); (iii) *ecl1* mRNA increased by 100- to 200-fold upon leucine starvation or magnesium starvation of leucine-auxotrophic fission yeast in a manner that depends on transcription factor Fil1 (17, 18); (iv) *ecl2* expression was increased only 2-fold by leucine or magnesium starvation, whereas *ecl3* expression was unaffected (17, 18); (v) *ecl2* mRNA was rapidly upregulated by 4-fold during heat shock, whereas *ecl1* mRNA was unaffected and *ecl3* mRNA declined by 5-fold (19).

*ecl3* is expressed at a low basal level during vegetative growth and little was known about physiological cues that induce its expression or the transcription factors involved. Our RNA-seq analysis of phosphate-deprived *S. pombe* (7) showed that the *ecl3* transcript was upregulated by 170-fold at 4 h, 300-fold at 8 h, and 400- to 440-fold at 24 to 48 h of phosphate starvation (Figs. 4 and 6B). *ecl1* expression was unaffected (<2-fold change) during the 48-h period of phosphate starvation, whereas *ecl2* mRNA decreased by half at 36 and 48 h (Fig. 6*B*). *ecl3* expression did not change during the switch from YES to ePMGT+PO_4_. Thus, *ecl3* transcription is specifically and selectively responsive to phosphate status and not to the several other nutrients that affect *ecl1* (*e.g.,* sulfate, leucine, magnesium). Simultaneous with our report (7), Ohtsuka *et al*. (20) also found that *ecl3*^+^ is induced by phosphate starvation.

We find here that full induction of *ecl3* during phosphate starvation relies on transcription factor Pho7, absent which *ecl3* RNA was more modestly induced and with a temporal delay (26-fold at 4 h, 45-fold at 8 h, 68-fold at 24 h, 107-fold at 36 h, and 180-fold at 48 h of phosphate starvation) (Figs. 4 and 6B) compared to WT cells. The *ecl3* gene is located on chromosome II, adjacent to and in opposite orientation to the *prt2* lncRNA gene of the *prt2*–*pho84*–*prt*–*pho1* gene cluster of the *PHO* regulon. *ecl3* RNA is coregulated with the Pho7-dependent *pho84* and *pho1* transcripts in a variety of fission yeast mutant strains under phosphate-replete growth conditions (21, 22, 23, 24, 25, 26) (Fig. S3). Whereas expression of *ecl2* remained unaffected during phosphate starvation of *pho7*Δ cells, the *ecl1* transcript was upregulated at 8 to 24 h of starvation by 8- to 11-fold (Fig. 6*B*).

### The *ecl* gene family collectively prolongs the lifespan of phosphate-starved fission yeast

The Aiba lab concluded, *via* characterization of single *ecl* deletion mutants and a triple-deletion mutant, that the three Ecl paralogs play functionally redundant roles in prolonging the chronological lifespan of cells during stationary phase, particularly in the absence of sulfate, leucine, or zinc (14, 16, 17, 27). Here, after a preliminary experiment showed that an *ecl3*Δ strain we constructed did not differ significantly from WT with respect to survival during 4 weeks of phosphate starvation, we proceeded to construct an *ecl123*Δ triple mutant. The instructive finding was that simultaneous absence of the three Ecl paralogs shortened the lifespan of phosphate-starved fission yeast, leading to reduced viability after 4 and 7 days of starvation (27% and 13% survival, respectively) (Fig. 6*C*).

### RNA-seq analysis of phosphate-replete *ecl3*Δ and *ecl123*Δ cells

A role for the Ecl family in gene expression was suggested based on a microarray analysis of logarithmically growing fission yeast cells in which the *ecl1*, *ecl2,* or *ecl3* gene was provided on a high-copy plasmid (28). Ohtsuka *et al*. (28) identified 65 coding genes that were upregulated by more than 1.7-fold when any of the three *ecl* genes was present in high copy, including the *PHO* regulon genes *pho1* (up 7- to 12-fold), *pho84* (up 3- to 4-fold), and *tgp1* (up 4-fold). The microarray study identified nine coding genes that were downregulated by more than 1.7-fold when any of the three *ecl* genes was present in high copy. Because the effects of forced overexpression do not necessarily signify a gene regulatory phenotype under physiological conditions, we undertook to gauge the impact of Ecl3 ablation, either singly or simultaneously with Ecl1 and Ecl2, on the fission yeast transcriptome. We performed RNA-seq on poly(A)^+^ RNA isolated from WT, *ecl3*Δ, and *ecl123*Δ cells during logarithmic growth in YES medium at 30 °C. Three biological replicates were sequenced for each strain. We found that no transcripts (other than *ecl3* itself) were dysregulated by ±2-fold in *ecl3*Δ cells. Thus, Ecl3 *per se* does not impact gene expression during growth in rich medium.

In *ecl123*Δ cells, we detected 32 coding RNAs that were upregulated by at least 2-fold of which four were increased by between 4- and 6-fold. A set of 56 mRNAs was downregulated by at least 2-fold in *ecl123*Δ cells of which 11 were reduced by between 4- and 49-fold (Table S4). None of the *PHO* mRNAs were downregulated in *ecl123*Δ cells. We reasoned that if a gene is truly subject to regulation by the Ecl proteins, we would expect the gene to be downregulated by pan-Ecl deletion and upregulated by Ecl overexpression. By comparing our RNA-seq data for *ecl123*Δ cells with the earlier microarray study of Ecl plasmid–bearing cells (28), we highlighted 9 genes that meet this criterion—*mfm1*, *mam2*, *ste11*, *spk1*, *map1*, *mfm2*, *ste4*, *rgs1*, and *mam1*—each of which is involved in mating and pheromone signal transduction. By contrast, there was no overlap between the genes identified by RNA-seq as upregulated in *ecl123*Δ cells and those identified by microarray as downregulated in Ecl plasmid–bearing cells. These results implicate Ecl proteins as a positive influence on the expression of mating-related genes; whether this reflects a direct effect of Ecl proteins on transcription is not clear.

### RNA-seq analysis of phosphate-starved *ecl123*Δ cells

To gauge whether the Ecl family is involved in expression of the *PHO* regulon during phosphate starvation, we first assayed cell surface-associated Pho1 acid phosphatase activity of WT and *ecl123*Δ cells as a function of time after transfer from YES to ePMGT(–PO_4_) medium. Pho1 activity, normalized to cell density, increased linearly during the first 12 h of phosphate starvation and continued to rise between 12 and 48 h, albeit with a shallower slope. The induction profiles were virtually identical in WT and *ecl123*Δ cells (Fig. S4).

To probe how loss of Ecl proteins affects the global transcriptional responses to chronic phosphate starvation, we performed RNA-seq on poly(A)^+^ RNA from *ecl123*Δ cells harvested prior to (time 0) and 4, 8, 12, 24, 36, and 48 h after transfer from YES to ePMGT–PO_4_. (The Bioanalyzer profiles of the total RNA samples from *ecl123*Δ cells indicated that the large rRNAs underwent fragmentation during the starvation time course akin to that observed for WT and *pho7*Δ cells [not shown]) Three biological replicates were sequenced for each time point. Here again, a cut-off of ±2-fold change in normalized transcript read level compared to the time 0 control read level and a *p* value of ≤0.05 were the criteria applied to derive an initial list of differentially expressed genes as a function of starvation time. Genes differentially expressed at two or more time points were deemed responsive to phosphate starvation. The findings are summarized as follows. mRNAs encoding 22 proteins driving autophagy were upregulated during phosphate starvation of *ecl123*Δ cells (Table S5). mRNAs encoding 22 enzymes/proteins involved in phosphate dynamics were upregulated (Table S5). mRNAs for 21 Pol2 transcription factors were upregulated (Table S5) *versus* 26 in WT cells (7) (overlap = 13). By contrast, mRNAs for 47 proteins of the 60S ribosome, 35 proteins of the 40S ribosome, 116 ribosome assembly factors, eight subunits of the Pol1 transcription machinery, 29 tRNA biogenesis factors, and 45 translation factors were downregulated (Table S5). mRNAs for ten constituents of the Pol3 transcription machinery were downregulated, while the mRNAs encoding TFIIIB subunits Brf1 and Bdp1, and the Pol3 repressor Maf1 were upregulated (Table S5). Thus, the major transcriptome changes in phosphate-starved *ecl123*Δ cells were similar to those seen previously in WT cells (7).

A noteworthy finding, not commented on previously (7), was that mRNAs encoding 11 aminoacyl tRNA synthetase (aaRS) enzymes were downregulated during phosphate starvation of WT cells; proteomics analysis affirmed that the levels of 11 aaRS proteins were reduced by 2- to 3-fold after 24 h of phosphate starvation (7). Whereas we found here that ten aaRS mRNAs were downregulated in *pho7*Δ cells, phosphate starvation elicited a greater effect on aaRS gene expression in *ecl123*Δ cells, whereby 25 aaRS mRNAs were downregulated by 2- to 8-fold (Table S5). Dampening tRNA charging is of a piece with the general trend toward attenuation/alteration of protein synthesis as a key component of fission yeast adaptation to sustained phosphate starvation.

## Discussion

We reported previously that chronic phosphate starvation of fission yeast elicits G0 quiescence associated with coherent changes in the transcriptome and proteome (7) that we presume are adaptive for survival. Activation of the autophagy pathway is typical of the response to nutritional stress that allows for recycling of macromolecular components. Beyond activating the autophagy pathway (29), phosphate starvation of fission yeast triggers increased expression of a large ensemble of autophagy genes (7). The factors governing transcription of the autophagy genes during phosphate starvation have not been defined, but there are preliminary indications that at least two separate regulatory schemes apply, based on the effects of inhibiting new protein synthesis during the shift to phosphate-free medium. To wit: (i) the starvation-induced increase in mRNAs encoding 11 autophagy factors (*fun14*, *atg43*, *atg1802*, *atg8*, *atg7*, *atg10*, *atg15*, *aut12*, *nbr1*, *nrf1*, and *scs22*) was abolished in the presence of cycloheximide, whereas (ii) the induction of mRNAs encoding nine other autophagy factors (*atg1*, *atg4*, *atg1801*, *atg20*, *atg2402*, *atg101*, *atg13*, *atg6*, and *isp6*) was not affected by cycloheximide (7). Here, we found that genetic ablation of key autophagy factor Atg1 (a serine/threonine protein kinase) results in early demise of phosphate-starved fission yeast. Though not surprising, this observation is in keeping with the idea that autophagy-driven catabolism dampens metabolic demand and provides life-sustaining metabolites.

Fission yeast respond to acute phosphate starvation by alleviating the lncRNA-mediated repression of a three-gene *PHO* regulon that encodes a cell surface acid phosphatase (Pho1) and transmembrane transporters of inorganic phosphate (Pho84) and glycerophosphocholine (Tgp1) (1). Synthesis of the three *PHO* mRNAs is driven by the Zn_2_-Cys_6_ family transcription activator Pho7 (2, 3, 4, 5, 6). The present transcriptomic analysis of phosphate-replete and phosphate-starved *pho7*Δ cells affirms and extends earlier microarray studies conducted by Wykoff and colleagues (2). We see that Pho7 contributes in several ways to the phosphate starvation response: (i) as a transcriptional activator of genes involved in phosphate acquisition and phosphate mobilization, not limited to the original three-gene *PHO* regulon and (ii) as an activator of additional starvation-induced genes with no clear connection to phosphate dynamics. We draw a connection between Pho7-dependence of gene induction during phosphate starvation and the presence of candidate Pho7 DNA-binding sites (4, 5, 6) in the promoter regions of the Pho7-dependent genes, the implication being that Pho7 is directly driving their transcription. Pho7 is distinguished from most other fungal Zn_2_-Cys_6_ transcription factors in that it binds to an asymmetric DNA target site and does so as a monomer (4, 5, 6). The 738-aa Pho7 protein is predicted to be almost entirely disordered, except for its DBD (aa 279–339). Outstanding issues regarding Pho7 function include: (i) how the protein segments flanking the DBD direct the Pol2 transcription machinery to Pho7-responsive genes; (ii) whether distinct disordered regions within Pho7 specialize in regulating specific subsets of responsive genes; and (iii) whether Pho7 collaborates with one or more other DNA-binding transcription factors (*e.g., via* heterodimerization) to activate its target genes.

The RNA-seq results also implicate Pho7 as a subtle contributor to the global downregulation of the protein synthesis machinery during chronic phosphate starvation. mRNAs for 44 ribosomal proteins and 85 ribosome assembly factors were downregulated during phosphate starvation of *pho7*Δ cells, which represents an attenuated response compared to the 129 ribosomal protein mRNAs and 115 ribosome assembly factor mRNAs that were downregulated in WT cells (7). Depletion of ribosomal protein mRNAs during phosphate starvation of *pho7*Δ cells correlated with the stable fragmentation of 18S and 28S rRNAs, as noted previously for starved WT cells (7).

We found that the absence of Pho7 resulted in shortened cellular survival during phosphate starvation. Although the magnitude of the *pho7*Δ effect on lifespan was similar to that of *atg1*Δ, we suspect that *pho7*Δ is not acting *via* defective autophagy, insofar as the autophagy genes are induced in phosphate-starved *pho7*Δ cells. It is conceivable that dampening of the ribosomal protein and ribosome assembly factor changes in *pho7*Δ cells might account for the effect on survival. Interrogating the functional consequences of ribosomal protein depletion and rRNA incision (*e.g., via* analysis of the abundance and compositions of polysomes and free ribosomes in phosphate-starved cells) will be essential to understanding the nexus between protein synthesis and chronological lifespan.

The *ecl3* gene, which is strongly induced during phosphate starvation (7), is located adjacent to and in opposite orientation to the *prt2* lncRNA gene of the *prt2*–*pho84*–*prt*–*pho1* gene cluster of the *PHO* regulon. The annotated *ecl3* mRNA transcription start site is 715 nt upstream of the *prt2* lncRNA transcription start site. We consistently noted in prior studies (21, 22, 23, 24) that *ecl3* RNA was coregulated with the *pho84* and *pho1* transcripts in a variety of fission yeast mutant strain backgrounds that either derepressed or hyperrepressed the *PHO* genes under phosphate-replete growth conditions (Fig. S3). Here, we find *via* RNA-seq that *ecl3* induction during phosphate starvation was squelched in *pho7*Δ cells. Similar findings were reported recently by Ohtsuka *et al*. (20), who measured *ecl3* mRNA by RT-qPCR during a 6-h interval of phosphate starvation of WT and *pho7*Δ cells. The *PHO* genes are turned on during phosphate starvation when synthesis of the upstream interfering lncRNAs is shut off (7), thereby allowing access of Pho7 to the *PHO* mRNA promoters. The *pho84* promoter resides within the *prt2* gene, which includes at least four Pho7 DNA-binding sites, as gauged by EMSA assays with overlapping genomic DNA fragments spanning 1100-bp upstream of the *pho84* transcription start site (30). One of the high-affinity Pho7 binding sites was mapped *via* DNase foot printing to a 12-mer sequence (^-977^TCGGTCTTTGAA^-988^) that adheres to the consensus Pho7 recognition element (4, 30). A parsimonious model is that shut-off of the *prt2* lncRNA promoter allows access of Pho7 to one or more of its binding sites in the *prt2* DNA and that this binding then activates the *ecl3* mRNA promoter. We reported that the upregulation of *pho84*, *pho1*, and *tgp1* during acute phosphate starvation was quashed by cycloheximide treatment, owing to a failure to shut off the production of the upstream interfering lncRNAs (7). Though not explicitly mentioned previously, it is noteworthy in the present context that induction of the *ecl3* transcript during acute phosphate starvation was also interdicted by cycloheximide (7), consistent with a scenario in which *prt2* lncRNA synthesis interferes with expression of both *pho84* and *ecl3*.

Our characterization of an *ecl3*Δ mutant reveals that Ecl3 *per se* plays no apparent role in fission yeast gene expression and has no impact on cell survival during phosphate starvation. Aiba and colleagues have implicated the Ecl1, Ecl2, and Ecl3 paralogs collectively in cell survival during various nutritional stresses *via* short lifespan phenotypes associated with an *ecl123*Δ triple deletion. Here, we present evidence that pan-*ecl* deletion curtails lifespan during chronic phosphate starvation. Transcriptome profiling of phosphate-starved *ecl123*Δ cells uncovered no obvious “smoking gun” to account for their accelerated demise. Rather, the gene expression changes elicited by phosphate starvation of *ecl123*Δ cells were much the same as those observed for WT cells. In particular, we see that phosphate-starved *ecl123*Δ cells mount an induction of autophagy genes *à la* WT cells. This observation contrasts with the effects of pan-*ecl* deletion on the fission yeast response to sulfate starvation, whereby the 3- to 4-fold sulfate starvation-induced increase in mRNAs encoding autophagy proteins Atg1, Atg3, Atg4, Atg8, Atg13, and Atg20 was effaced or attenuated in an *ecl123*Δ strain background (31). A potentially interesting difference was that phosphate-starved *ecl123*Δ cells experienced a more widespread downregulation of mRNAs encoding aminoacyl tRNA synthetases vis-à-vis WT or *pho7*Δ cells. How this phenotype might connect to the Ecl proteins is unclear.

## Experimental procedures

### Growth media

The recipe for 1 L of ePMGT medium contains the following ingredients: potassium hydrogen phthalate (3 g); anhydrous sodium phosphate dibasic (1.66 g); anhydrous sodium phosphate monobasic (0.46 g); glucose (20 g); adenine (0.25 g); uracil (0.25 g); glutamic acid (3.75 g); histidine (0.25 g); lysine (0.25 g); leucine (0.25 g); thiamine (5 mg); 1000× vitamins (1 ml); 10,000× minerals (0.1 ml); 50× salts (20 ml); and amino acid mix (2.7 g). The components of the vitamin, mineral, and salt stocks are as defined previously (7). The amino acid mix is composed of alanine (2.8 g), arginine (1.3 g), asparagine (0.5 g), aspartic acid (2.65 g), cysteine (0.10 g), glutamine (0.1 g), glutamic acid (4.70 g), glycine (1.50 g), histidine (0.65 g), isoleucine (1.5 g), leucine (2.0 g), lysine (2.3 g), methionine (0.4 g), phenylalanine (1.3 g), proline (1 g), serine (0.8 g), threonine (0.8 g), tryptophan (0.25 g), tyrosine (0.60 g), and valine (1.75 g). The pH is adjusted to 5.6 as needed by addition of NaOH. Sodium phosphate salts are omitted from ePMGT(–PO_4_).

### Phosphate starvation

Fission yeast strains were grown at 30 °C in YES medium to *A*_600_ of 0.5 to 0.8. The cells were harvested by centrifugation, washed with ePMGT medium without phosphate, and resuspended at *A*_600_ of ∼0.3 in ePMGT(–PO_4_). The phosphate-free cultures were incubated at 30 °C and diluted with ePMGT(–PO_4_) to not exceed *A*_600_ of 0.8. At the times specified: (i) a volume of culture containing 10 *A*_600_ units of cells was rapidly cooled by introducing it into ice and cells were harvested by centrifugation at 4 ^º^C. The cells were transferred to 1.5-ml screw-cap tubes and then frozen on dry ice for subsequent RNA analyses.

### RNA-seq analysis

Total RNA was extracted *via* the hot phenol method from 10 *A*_600_ units of *pho7*Δ cells or *ecl123*Δ cells harvested prior to (time 0) and at 4, 8, 12, 24, 36, and 48 h after transfer to ePMGT(–PO_4_) medium or from WT cells harvested prior to (time 0) and at 2, 4, and 8 h after transfer to ePMGT(+PO_4_), or from *ecl3Δ* cells that were grown in liquid YES medium at 30 °C. The integrity and quantity of total RNA were gauged with an Agilent Technologies 2100 Bioanalyzer and TapeStation. The Illumina TruSeq stranded mRNA sample preparation kit was used to purify poly(A)^+^ RNA from 500 ng of total RNA and to carry out the subsequent steps of poly(A)^+^ RNA fragmentation, strand-specific complementary DNA synthesis, indexing, and amplification. Indexed libraries were normalized and pooled for paired-end sequencing performed by using an Illumina NovaSeq 6000 system. FASTQ files bearing paired-end reads of length 51 bases (total paired reads of 14.9 million to 37.7 million per biological replicate) were mapped to the *S. pombe* genome using HISAT2-2.1.0 with default parameters (32). Mapped reads comprised 89% to 99% of the total reads per replicate. The resulting SAM files were converted to BAM files using Samtools (33). Count files for individual replicates were generated with HTSeq-0.10.0 (34) using exon annotations from Pombase (GFF annotations, genome-version ASM294v2; source “ensembl”). Reads per kilobase per million mapped reads analysis and pairwise correlations (Pearson coefficients of 0.963–0.989) were performed as described previously (35). Differential gene expression and fold change analysis was performed in DESeq2 (36). Cut-off for further evaluation was set for genes that were up or down by ≥2-fold at two or more starvation time points compared to the WT time 0 control, with a *p* value (Benjamini–Hochberg adjusted) of ≤0.05. Genes were further filtered on the following criteria: (i) ≥2-fold up and the average normalized read count at a given time point was ≥100 and (ii) ≥2-fold down and the average normalized read count for WT time 0 was ≥100. This served to cull poly(A)^+^ transcripts that were present at very low levels in all samples. After deriving a list of differentially expressed genes for each time point, all genes that did not have a 2-fold change in expression at two consecutive time points were culled.

### *S. pombe atg1*Δ strain

Genomic DNA segments (619-nt upstream of the *atg1* translation start codon and 618-nt downstream of the stop codon, respectively) were PCR-amplified and cloned upstream and downstream of the *hygMX* antibiotic resistance cassette in a bacterial plasmid. The linear *atg1*Δ*::hygMX* gene disruption cassette was excised from the plasmid and transfected into diploid *S. pombe* cells. Hygromycin-resistant transformants were selected and analyzed by Southern blotting to confirm correct integration at the *atg1* locus. Confirmed heterozygous diploids were sporulated and hygromycin-resistant *atg1*Δ haploid progeny were selected.

### *S. pombe ecl*Δ strains

PCR amplification and standard cloning methods were used to construct plasmids in which an antibiotic resistance cassette is flanked by 532- to 610-bp DNA segments corresponding to genomic sequences upstream and downstream of the *ecl1*, *ecl2*, and *ecl3* ORFs. The resulting *ecl1Δ::hygMX*, *ecl2Δ::natMX* and *ecl3Δ::kanMX* disruption cassettes were excised from the plasmids and transfected into diploid *S. pombe* cells. Transformants resistant to hygromycin, nourseothricin, and G418 were selected and analyzed by Southern blotting to confirm correct integration at one of the *ecl* loci. Antibiotic-resistant *ecl1Δ*, *ecl2Δ*, and *ecl3Δ* haploids were isolated after sporulation of the heterozygous diploids. Serial pairwise matings of the *ecl*Δ strains and random spore analysis with selection for two or three drug-resistance makers inserted in lieu of the *ecl* ORFs yielded an *ecl123*Δ triple-deletion haploid strain. Strain genotypes are shown in Table S6.

## Data availability

The RNA-seq data in this publication have been deposited in NCBI's Gene Expression Omnibus and are accessible through Gene Expression Omnibus Series accession numbers GSE247799, GSE247802, GSE247922, and GSE247924.

## Supporting information

This article contains supporting information.

## Conflict of interest

The authors declare that they have no conflicts of interest with the contents of this article.
