# Supplementary material for: Factors governing the transcriptome changes and chronological lifespan of fission yeast during phosphate starvation
Source: J Biol Chem. 2024 Feb 2;300(3):105718. doi: 10.1016/j.jbc.2024.105718 (PMC10910108; doi:10.1016/j.jbc.2024.105718)
Supplement: Supporting Information [file mmc1.pdf]

Supporting Information

Factors governing the transcriptome changes and chronological lifespan of fission yeast during phosphate starvation

Angad Garg, Ana M. Sanchez, Beate Schwer, and Stewart Shuman

Figures S1, S2, S3, and S4

Table S6

Tables S1, S2, S3, S4, and S5 are separate .xls files

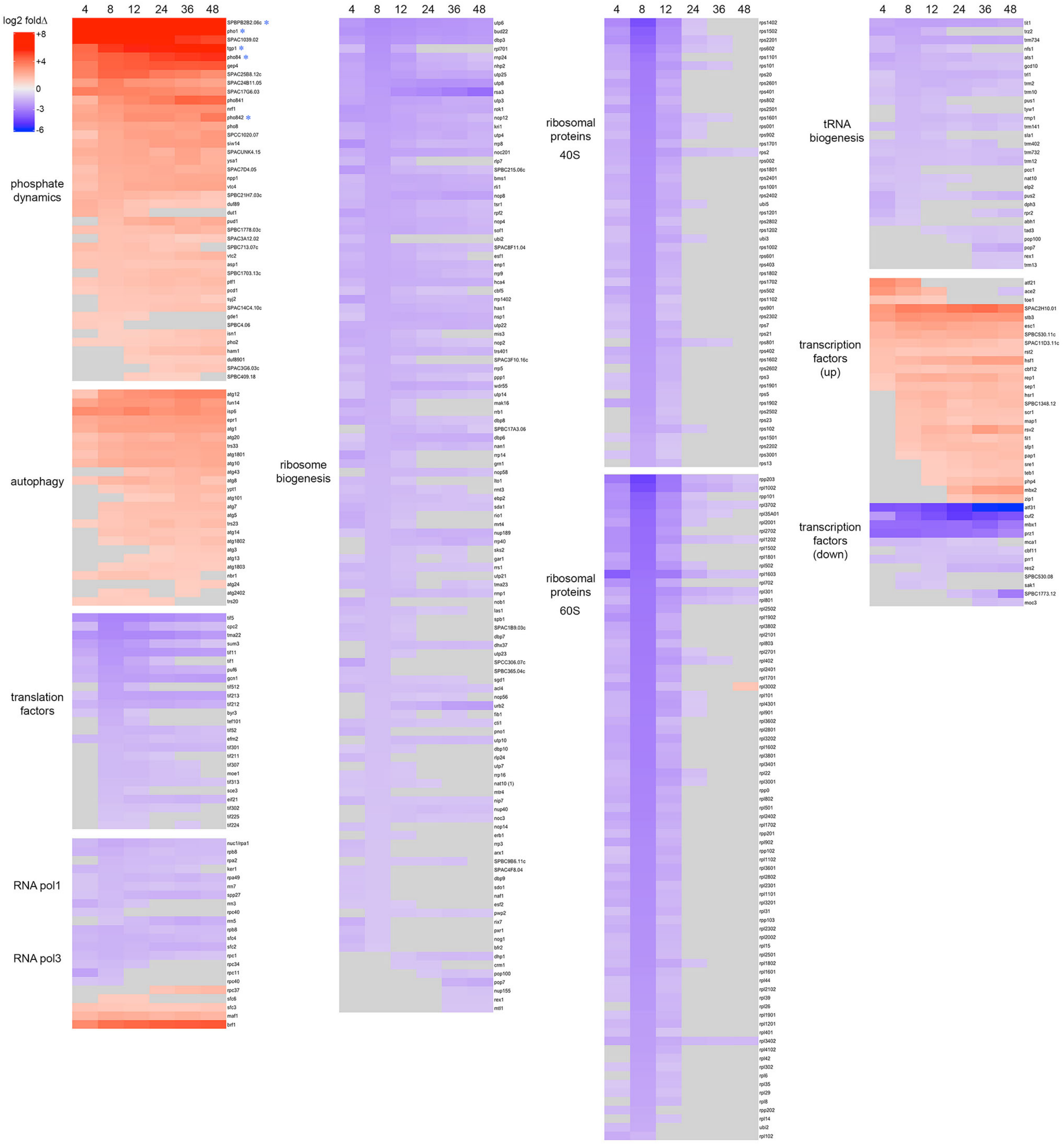

Figure S1. <u>Phosphate starvation-induced changes in mRNA expression in wild-type fission yeast affecting the indicated functional classes of genes</u>. The RNA-seq data used to generate the heat map are from ref. 7. Each row represents a gene (specified at right), and each column represents the duration of phosphate starvation (h). Pho7-dependent genes are denoted by blue asterisks.

Figure S2. Pho7-dependent genes with upstream Pho7 binding sites

Key:
5′ flanking DNA
Pho7 site
TATA-box
Transcription start site
5′-UTR
Open reading frame
Intron

str3 plasma membrane heme transmembrane transporter
TCAAACTATACTCGACTTTA**TCGGATTAATAA**ATTAGCTAATCAGTAACCAGGGTTGTCTTGGCATCGGCATATCACTGT
ACAAAGAAGGAAATTGCGAATAACTACTAACTAAAGTGGAGATCTCATTTGTCAAAGTCTTCAATTCGAGCTTCTTAACC
GCGGTGCCGTTTGGAGTTTTGTCAAAATAAGGCAAAAACTTTTCATAATCCGAACCATGATCTGATATGCGATTTAAGGT
TAAGCGTCCATAAAAGCATATAAAAGAAGTTGTGCATCCAGCGAAGCAATT**G**CATATTATTTTGTTTACTATTGAATAAT
CATGGAAGCAAAAGAAACACATTCTATATCAGATCATGAAGTTGAATTGCAGGATGCAAAGCCGGAGGAGAAAAGCGAAA
ATGGAAATTTTGTCTTTGAAAAGGCTTTCTCTTCT

SPBPB2B2.06c extracellular 5'-nucleotidase
TTTTAGGACAACTT**TTTGTCAACCGA**ACATAGCGTT**ATTATATACCGA**AGGACGTAAATGACATAATGCAAAACACAAAT
GTTCCATTAACAGTATTTTGAGACTGATGTTACGGTTTTCCC**ATCGAACTCCGA**GTCATTCCCATTGCTAAAGAGTTTTT
TTACTTAAATAAATGTTTGAGTAAGGGAACGAAGAAATAACACTGTCTATAATTATTATGAATTTTGATTGTTTAAATTA
ATATAATGTAAGAAGATACATTTGCAGCAAACTCAAAGTTGCTTCGCAACTTCAATTCATTACTAAATTTTGACATTAGG
TCATTAGCAAAATGGGACTGAAATCTCCGACAAAGTTTTGTAAACCTTTTTTTGACACTGACACTTTTCAAAGGTTGTA
CGATGTTAATGAATTGACTGAGGGAAATTTGAAAACATTGAATT**TCGAAATGCCGA**TCTTTTTTTGTATAAATTAAAGAA
TCTTACCCTCGGTAAACAGC**G**AAATAGATTGTTAAGCTCTTTATCCCGACTCCCTCAACATGAAGACAGCCTCGATACAT
TTTTGGTCAACCCTTGTGCTTTTGTTCAGTTGTATTGGTTCTGTAATTGCTTACTCGTCTACCGATTTAACTACAATGTA
TGATTGGAACCAAATCA

SPBPB2B2.05 class I glutamine amidotransferase
TAAAGACTACAATCCAA**TCGGCTATACAA**GGTAACAAAGTATTCAACTAGTTGCTCTTTTAGTCAAGGGATATTTTATAA
AATTCCTTTGTTTTCCTTAGCAACGCTAAAAATTTGTTTTGGGAAATTTGATAAGCAATCAGAACCTAGAGGTATCTCAT
AAGCATTTCTTCAACCAAATGCACTTTGTTTTAAAACATTTATAGACTTTAAAATATAAGTTTTACTACTGATCAAGCAA
TCATAAAAAATGAGTGCAAAGTTTAGAAAATTTAAGAGCGTTTTTTACGCTTCTTACCCATTGCCAAACAATTAAAAATA
AAGCTCATAACAAATAATAACGCTTTTTTCGCACGGATGATAGACAAGTCCCATAGTAGTGAATCTGTAGAACGGACGGC
AATTCC**TCGGAAGTTCCA**TGATCCCAGATCGTAAACCTTTTGACTATCTGCCGGAATCCAATCTAGAATCTCGTAAATAG
ATTTCCTATAATCTCCTGAATTTGCTACATTCCTTAACAAATCTTTTGCTAGAATTTTTATCTGGGTCTCTCCATTCTTA
GAGCACCAAAAACGCAGAAATAAACTAGAGTAAGGATATATAGCATATACATATATATATATATATAACCTGCTAAGTTA
TGTAGCTCGCAATTTCTCCACAGTATTGCGCAATTTTAGTGCAACAAGTACATGTAACTTATTGCTATCTGATTTTCTCC
TCGCTACTAATCAAGTCTGAATAAGCGAAAACGACTTTTTAAGCGAGAAGTAACTTATGCCT**TCGCAGTTTCAG**CATAAT
TTTCACTAGTGTGATAGTAATTCTAGAATGTTCAAAATTACTTTGGACAGAAACAAAGTCTCATATTTTCTGAACTCGTA
TAATTTTTTATTAATTCAATTTTTGTCATTCCCGCTTTCCGCAATTGGCTCATACCTTGAAACAATAGCGGGTCGTTGTG
CGTTTTTGAGGCATTTTAGTTGATACTAAATTAAGCGATAAGAAACCAATATATATATATCCAAGTATGGTGATAATTTT
AT**A**GCGAAATAGAAAAGATGGAATGCCCTATTATTGCTTTGAGTGTCGGGTTTTCTAATAACTCACAGCCGTATGTAGAA
GCAATTATCAAGGCAGGTGG

pmp20 thioredoxin-related chaperone
ACAACA**GTCAAATACCGA**ATATCGCACGAACCATTGAAAACGCAGCTGTGCGCGGTCTCAGTGCCGGTTGGGATTTTCCA
CCACTCGCAGCCCACAAAGCTCTTCATACAAAGATGAAGAACAGTAAAGAAAAAAAATCTTCCATCCCGACAAATTTGGT
GATTTGACGTCACCAAAAGATTCGTTTTTGTAGAAAGGTATATATATTAACGACATGTGCCCATTTGTCTT**G**AT**A**ATACT
ATCTTTAAACTTGAACGACATTTTTTGAATACGCCGTTACTCCTAAAGATTTTATACCACTTGCTTTAACTTTTACACAA
ATTTATTACTACTATTAAAAATGGTTGCTGTCGGATCTACTTTGCCTAAAGTTACTCTTTGGGAAAACAAGCCCGAAGAG
GTTGTTGAATTCCCTTCTCAAGGCAAATTCATCATTGTCGGTGTTCCCGGCGCTTTCACTCC

mfm1 M-factor precursor
TATTAACAATTGACTAGA**CAATGGGTCCGA**CCAACAAAGAAGTCTCAGTTTTTTTAAAACTTGGGAATTAAAATTTGTA
TTAAAGGACAGGATAATTCTGTATTTTCGCC**A**TTGAATTCAACAGCTTTCAATTCACTCTCACGTCTCTTAAACCTTTTT
GTTGACATTTCGTTTTTCACTTTCTTTACTTAACAATGGACTCAATGGCTAACTCCGTTTCTTCCTCCTCTGTCGTCAAC
GCTGGCAACAAGCCTGCTGAAACTCTTAACAAGACCGTTAAGAATTATACCCCCAAGGTTCCTTACA

gpd3 glyceraldehyde 3-phosphate dehydrogenase
AAGGCCGCTAAT**TTCATTATCCGA**TCTGCCAAGTACCGAACACGGAAATCGCCGCTTACGATACCGTCGTTGGCATGACA
ACTCTAGCGTAAACACATACAATATAAATAGGAGTGGTAGGCCAGTCTGCTTCTTT**G**TAGTATCAAGAAATCAGCAATAG
TACTTGTTTGGTTTTGAGAAATAGCAATCGAATTAATTAG*GTATGTGATGCGCTGTGGCATTGGCAAAGCATTGGCTTGG*
TGAGACATGTGACATGTGTCAAACACCTGCCAAGAGGGAAAAATAGATTGAGGAAAAGGAAGGGATATTTTCTTTTGATA
TTCTCTTTTAGTTGAAAAAGATGGATTTTGAGCGATGAATTGAAATGAAAATGGTCAGTGAGATTGCCAAGAGAAGAGGC
TCTCTGATTTTACTTGACATGACGAAAACATGTCATATCCAGTATGGTGAAATTGTACAACGAAGTCGTGATAAGATTCG
TTAGTTTCCGTTTCTTTCGGTAAAGAAGGCTTTATTTTTCGCTTGACGGCAGTTTGGTCAGAAATTAAAATAGACTTGCC
GAAAGCGACAAAGCTGCTGTTTTTAGTATACTTTCTTTTCTATTCTTCTCTCAATAATGATATCTTTTCTTTTAATGACG
CTCATCGTTTCTTCTTCTTCTTCTCTTCGCTTTCTTCCCTCTCTCGCTTCTTTTTGCCTTTTTCTCTCCTCTTTGATCTC
*TTCTTCTTACTAACTCTTTTCAG*ATAACAATGGCAATTCCTAAAGTTGGTATCAACGGTTTCGGTCGTATTGGCCGTATC
GTCCTCCGTAACGCCATCCTCACTGGCAAGATCC

sro1 stress responsive orphan
TAGCTTACGATTGCTTTCGTCTAGGTA**TCGGACTTTACA**CTGCTAGTCGCCTATTTTAACGTTGCGCCGTATGGAACGGG
TATCGCATTCTTATCAGTAAGGAAAGCTATCTGAATTCGTCACAAAGGAAAAGGCAAATAAATAGTCTAAAACTATATAA
ATGCGGGCTTCCATCTAAGAGACCTC**G**CCATCCAACAATTGTTGCGTCCCACCTAAACAGTGTCTAATCAAAGTCAATTG
CGAGACATTCTAAAGTAAAAAGTTAATACATTTTAGCTACTACTAAATACTTCTTTTGATATTCTTTTTAGTGAACAATA
AAGAATATTGGTTTGATCAACACGGTCATCTTCAGAAAAAAAATTTTTTATTCGTTGGTTTAATTAGTTAAGAAAATTTT
TTTTTTCTTTCTTCGCTGACGTCTCGATAATTAAATCTGCGAACGGTGATACTTCTATTCTACACATTTCTATTTAATCA
CTGTTATTTTCTTTTCTTTTTAATTTC**A**TCAATAAACATTTTTGATTCATCGCTATGTTTTCTATTCTTAAACAAGGTGT
CATCTCCAAGTCTCTTTTAGCCGCTACCTCTTTAAAGGCAACGGCTTCAAGATTTTCCTTCTCTACTAGTGTTGCTTCCC
GTGTTGACGGTACTAAGAA

SPAC4H3.08 3-hydroxyacyl-CoA dehydrogenase
TTATGACATAGAAACGTAAATG**GATGTTCTCCGA**CAAAAAATAAAAAAAATATATTATATATAGAGATAGACGTGCGCCT
TAATATATTTCC**A**ATTTACTACGACTGACTAACTAACTAAATAACTGCGACTTTATTAAAAAAACATTAATAAAATGGAT
CCTGTTCCTAGCACACAAACTCAAAAATGGCCTGGCAAGCATGCCGACTTAGATCCTGAACCATCACTTCTCAGATATTG
TGATGGACGGGTACATGTCGGATCAGGGAAACTTGCAGAGAAAAAGACATTGCTC

atd1   aldehyde dehydrogenase
GCGAGTTTGGGATTT**TCGGAGAGCGAA**AGAACATGGGGTTTCCTGCGAAAATGACCCATTTTGACTAGATTAGCCCAAGG
TGCTCCCATTTTCTCAGCGCATCGCATTCTTTGGGAAATAAGAGAAGGAAGAAAATTTAAGAAGCGAAAGCGCACTATGC
AGATGGCGCTAGAAGTTGCTTTGCTTTTTGCTACTAGACTAGACTAGGTTGGCTGGCA**TCGGAGTTTCAA**CGTCGTTCGT
CTTTCATCGTTCCTCGTTTGTTAATATCAAATTTTCGATATAGTGAA**TTGTATTTCCGA**GTATGCAATTCCCTCTAAAGC
ATATTACTATTGCACAGTGAGATTTGGGAGGGAAGCTTTGCGTACGCTAGCACATATGAAATCTTGTGATTTTTGAGGGT
GTCTCTTCCTTTGATTCGCCAAAATTAAGCATTGACATCATGGGGTAAGGTGTCCTGTTTTGTCATTCGTCGTTGTTTGA
ATTAGATCATGTATAGGAAATCATTCGTCCAGGTGTCGGTCTTTTGTGTCACCATTAATCAGTAGGAGTTGGAGCTTTGT
TTACTAATCATCGTATAAGTCATATATGATTTTTACTATAAATATTGAATTCGCCCCCCTTTGCAGTTC**A**CTGAAATAAG
TAGAACAGCACGGTCTTTTCGTTTCTACAACACGCTTGCTATTCTCTTATTAGTATATTCCTCTATCTATAGTCTTTCCT
TCACTTCCTACATCCAAATTGATATAGGTTTCCTTTGTGGTATTATTACTAACAACGACAATTGATTGTTTTAATTCATT
GATTTTGTCGTCTTTCGGTTTTTATCACATTTTATAAAAACTGCATCCCCCTCTTCTTAAGAATAATAAGAGGCCATTGT
TTATTTACCCCGGTCCTTTTCTGACCATCTAATCTTTTCGTATTAGGTGCTCTTTTACTTATAGTTTGGTTTCGCCCATA
ACTTTTTGACGACGTTGTTTACTTTGGTTGATATAAAATTATTTTATACAATCGTTAACACCTCATATTTTCCATTTCAT
TACTGATCTTTTGGTTTTGTCCTGTGTTTGCATTTCAATTCAGCTCTGCCAAATTTATCCCACTACTTCATTTTTTATCT
TTGGTTTTTTTTTTTTTTCTAGTTAATTTTTTAATTACTCTTACTCATTTACGACAACGACCCCTTTTCTCAATTTTTTAA
TACAAACTTTTCAAAATGTCTACAAAACTCGTTGATCACGTCGAAATTACCGTTCCTACAGGAAAAACTTACATTCAACC
TGTCGGTCTCTTTATCAATAACCAACATG

pho842 inorganic phosphate transmembrane transporter
TTTAACTTTTATCTTTTTCG**TCGGCCTATTTA**ACATTGAGAGATATAATTTATAGTTTGGTAGCTTTGGACCAATGGATC
GAGTGTTTGTTTACCGCTTTTGCGCTGCGACTCCGTGGCGAAAGTGCGCTTTTTGGATGATTGTCAAGTGTGCCATTTTC
GACGTAATAATTGGTGAATGTTAAATGACTAATATGTTACATTGAG**TCGGTCTTTGAA**AATCAACTGTAACAAGCGCCTA
AGCATCTTCAGGCAAAAACACTTATTATTTCTTCCTATATAAGTATTGCCTCTGCCGTTTGTCTCAGC**G**TGCTTTCACCA
GTTTGCTGCTTTCGCTATATAGGCAGTCAATTCATCCATAAAAATAAATTCGACTTGATTCGAAATTGCATTTCATCCCA
TTCTCAATTTCTTTCTCTCCTTTTTTGAAGGCAATTGTCGCTTTAGTTATTACATTTTTTGTTGGAGAACTCACTGACTT
CGAATAATTCTTTTTATTTTTCTCTTTCTCCTTTTTTTTTTCTTTTCCTTTTTGAAATCTGTTTTTCTTTTCTAAATTCT
ACAAATCTTGACCGAATTGTTTCAAAAACCTTCCATTTAATCTGTACATTCTTTCTCTTTGCAGGATTTCCCTTTTTCTA
TATATAATTTTTTTCTTATTGCAGTTGTTGAAAAACTTTTCTCCCCAATTCAACTCAATTCCTTGATTTGCATTCCTGTT
AAACAATCTATTCACTACACTATGAATCGCTTAAATCCTTTTTCAAAATCTCACTCCAAAAACTCCAATGAAAACAACGA
AGTTTCCTTGGCTGATGTCGCCGAGTCCGATACTCGTCGCCATTGGC

rgs1   regulator of G-protein signaling
TTCTTTTTTTATTATTATTCAA**TCGGTGTTATCA**CAT**GAAATTTACCGA**TTCATTAAAACCCCTT**TCGGAATAAGTT**GAC
GAAAGGTGGAAATTGCTACCTGTTTTCTTTGTTCTGCTTTCTCACTGTTAATTTGTTGGGGTAAACAAATCAATTGCACA
ATGTTCTTTGTTCCGTTAAAGTTCCCATTGTTCGCCATTTAATGATTTCTTTGTTTAAAAGAAAGCGTATAGCAATGGTT
AGATGGGAAACTTCGAAGTATAAATACTAAGGCATTTCCCGAATTTTCATC**A**GTGTGCCTATATCGACGTTTACAAAATT
GCTCCAATCCTTCATTCGATTTTAAATTTAATAGGGATCAGACCTATTGAAAACAGCTGGAGACCTGATTTAGTATCGAA
TTACCCGTAATTGATTATAAAAATTGACGGTAATCAAGAAAGTGGGACTATTACTATATATTTAAATTTCTTTTATCTTA
TTTGAGTTCTGTACTTCTCTTTTATCTTTTAAAAAAATAAAAGAAACAATATTTAACACTCCATTGTGCTTGGCTGCCCG
ATCTCTGTCGCTTTTTCGCTTAGCAAGATCACAATTCTTTTGTTCATCACTTTATTATTTATGATAAAAGGCTACAAATT
CGTAAAAGTAAGAATCAGCTTTGGATGCCCGCCCTTCATAACCCTTCTTCACCACCGCCCAGTTATGAAGCTGTTACGTC
TTATCGCAACGGAAATTCAATAGATTCCGGCGATAAACGTCAACAATGTT

cta3 P-type ATPase
GACTGGAATATGGGTCCTTTCTACTTCCATC**ACAAAACTCCGA**TCTTCGGTCGGTTCCCCATATTTCAGCAATTAAGTAA
CACATTACTTTCAAAGAAATAACATGTTACCCTTATCGTAATTATGTTACCT**ATAAAACACCGA**CATGTAGCCACCTTCG
TAATACTTATTAGCGTAAGGAGGA**TCGGCATATGAA**AATTGCAAAAGTATACGTAAACAATTAACACTGCAAATGACTAA
GCCTCAATCTTGGCAAACGTTTCAACGTTCAGGTTGCCTCTTGGATTTCATTCCTTGGTTAGTTTCTCTCAAAATTTCTC
GGTTTTTTTGACATTTTTTTTTCACTCTGAGTATTCTTCTCCTCTGTCATGGAAATCCACACTTCTTC**TTCTTTAACCGA**
ATAATCTTAAATGCTAAATCGTCCTCTCATCTTCGCTTCCCTTGAATAACAACACTCCGCCATTCGTCATTTGTTACTCC
GCTTGATAATGTCAGCTACTATGATTTACGTAATTTTATAGTTAAATTCCCCAGGCAAGCTTTGCTGCTTATTGTATCTC
TAGCCAATGTATGTTGAGGAAATCAATGTCGGAGTC**ATTGCAATCCGA**CACTCTGATATCGTTTTTCTTCCAAGCCGTAT
ATATACTGCTGCTATTCCTCTCACTTTTCTCT**A**TATAAAATATTCTTTAACTAAAAAGTAGTAACTCGTCCTTTTTTTTT
GAACATTTACTACCTGCTACCTGATCTGAGCTAAAGATCTGATAGCCCCTTGACGAAGATAACATTACGAGCTTCGGTTT
ACCGTGTTAAGTAAATTAAGCTCCCCTGATTATACTTTAATTATCACTCATTTTTCAAATTCTCTCGATCCCCTCCATTT
TAGTTTAACTCTTTTTTTCCCTCTTCAAAATTCCCCTTTTCGTTAATACACCTTCTTGCGGTCTTTTCTTTTTTCCGTCCT
GTGTTTATTTGTTTATTTGCCTTTCCACTTTTTGTGTTTTGCTAGATCTCTGTATTTGGATTTGCGTAGTACGCAATTAA
GTGAGCTCGAGTTTAATTTTAAAATAATACGTTAATTCCCCAAAAGGATCTCATTTGATCTGATCTGATCTGCTGTCCCT
TGTTGATCCTTGACTTTGCGTTTGGTTTTTGAAAACCCACAGCACAATTTGGTATTTTCTTTCTTTCTTTACCTTTTCGC
ATTCTAATTATTATTTCATTACACTCCCTACGCCTCTATAATTTCTGTTTACTGAGCGTGCGTGTGAGAATATTTGTGTG
TGACCTTGTTTACATCATCTACACGATTTATTTACTAATTTCGCTTCCAATCTTCTCTGCTATTTTTAGGTCAAGCATCG
TTCCATTAATTGTTTATATACATCTTTGAGCAATTTATTTTTAAAACAAAAAGTATTCCTCTAACTATTCCTATTTCCTC
CTTCTCCTCACTACCGCTTTTTTACTTTTATATTCAGACATAAACCTTTTCCTTCCTACATTTCTTTTGGAAAACAAAAC
CAGTTGCTTTTTTGATTTTACTTATATTTCTCCCCTTCTACTCATCCCGATATATTCTTACTTCCTTGATTCAATCTCAA
ATATTGTTCAATGGTAACCATTAATATCTCGAATCCCGTTTATTTCTCTGACATTAAAGATGTTGAATCAGAATTCCTTA
CGTCTATCCCCAAT

SPAC27D7.11c But2 family protein
GAAGTGCGACCAATATTACCTCTGCATCGCCAACAG**TCGGACTTTCTT**GTATGCGCTGACCATTTATTATGTAAATCATT
CACC**TCGCCTTAAAA**ATATATTACTACTCCTCATTTTAATTACCGTGTATGATTCTACATGCAATTTTTGTTTCTGCGCT
TTTCCCTACACATGACAAAAGTTTTCATGACTGCGGATCATGAGTT**ACGTTTTACCGA**AGTAAATTCTTGATATTGTCGA
ACTTAAGTTTGGAAAATTTTTGATATATAAAGAGATGAAAATCCTTTTAATATC**G**CC**A**ACAAATCTAATTGCATTCATTT
GTAATAATCTTCGAAGCCTTACTAAACAAAGTAAATAAGTCTTCATCAATTCCCTTTAATCTCTCATTATAATTATTATT
ATTATATTAACAAAACCTCCATATCTTTGCGAGGACTTTTTGGTTTATAAAAAACAGTTAATTAATTGGCATCTATTCAT
TGAAAATCTTGGATTTTTATTACACGCACCATGAAGTTTTCTTCGATTCCCATTGCTTCCACATTACTCTCTTTGCTCGT
TGCCTCCAGTGTGACAGCTTCCCCATTGCGTAGACGCGATGACCCTTTGAACGGCA

SPBC25B2.08 Schizosaccharomyces pombe specific protein

CCCAGTCACTGCTCAAGGTGTGCAAGTTACCT**TCGGTTCCTTAT**TAAATTGACATCAAAGACGGTTTGCAAATGTTACCA
GATATGGACGTATATATACATAGAATACCCCAATTAAGACTTT**G**AAATTCCGTCTTTCACTTTTTAGTCTATATCTATTG
TTCAGACTAAACGCAGGCTTAACGCCTTGTCCTACTCCCTTCGCTTCATCTTGTGCTACTCTATTGATGAGTATTTATTA
ATTTTTCCGCTTTCTTTTGATTAATTCATTCAGCGGTTTGTTTTTGTTTTTCCATCCTTTGTTGTCTTTCATATCGTTTT
ATTTCGTTCCTTTTTTTTTAACTATCTCAATTGGGATAAGCTTTATTTTTCGTAATTGTAATAAGCTCTAAGAAAACGTT
CCCTACTGTTTTACTTTTTTTTATTACTGTTCACTTTCTATTTCCTACTCACGGTTCTCTTGCTTTGCTTCCTTCGATAA
AACTAAGCAAGTTTGATCCTTTATTTATTCAATTGTCGGCTTCTAAAACTTCGTCTGTTCGCATAATTTCATATCTCGTT
AGGGTAACTTCTTTCGGACGGAACACTAAAGATTTCGTTTACTGATTATTTTCTGTTTACAGTCTGGACTTTATACCCAT
TTCTAAATAAATTAAACACCCGAAATATCACTACAACCCTATATCCCATTAATCCCTTGGCTATTTGGATTATCAAGCTT
ATTTATTATTCTCAGGTTTCTCTAATTTTGTGCATAATTCAAGTTTGAAAACATTTCGTGTAATTCAAAAAAAAAGTTTC
CCTTTGTTACATATTTGTCGAATTATTTTCTCATTAAAACCTTTAATCCCATCACTTCACCTTTGATTGTGATTTGTTTC
CATTTTACTTTTATTTTCTCTTTTTCTTAAAATTTATTACCATGCTTCCACATCAAAATTCCAGTTATACCCGTCAAGGC
ACTAATGATGCGCAGGCAAATGATATGCGCAGTCCATCGCAACTTCCTACTTCTGTCAACATTGAAGATCCCTCTAAGTT

ecl3 extender of chronological lifespan protein

GACAGGGTAAGATTGTGTACCGT**TCGGTATGCAAA**CTGAACTGGAAGATTGGAAAAATTAAACCCTTTTCTGAAAATAAT
AGGAATTAAGTTAGGATAGTCGAAACAATTTCTTCCATATAACTATCGTGATCAGTCAAGGGCAGTTTACTAATTTATTT
CCAGCGTC**GTATATTGCCGA**ATTCACACGCATTTTACTCACATCGTTCGAAGATAAAAAAAATATAAATAATAAAAATTG
AAATTCGTGTAAGCGAAAAGGAATGTGTGAGAAGGATTTTGGACTGTTCTATTCTCATGGGAATGTGAAGTAACTACTCT
CTTGCTACCATAGTTGCTTGTTGTGACTTGACATCGTATAAGTGACTGTAAATTAGATGCCGGCTCCATG**TCGCACAATA**
AACCTTTTGAGAGAAAATAAATTATGACATTCCTAAGAC**TCGGCAATTTTG**ATTTGTTATTTTTTTTTATTCTCTCAACA
TTTCCCTAGATTCACAGTCGAGAATGATACGTTGATACACATCCTTTCACGGTAATCTGCAATCCCTGTTTATGGTTTTA
CATCGTCCTTCGTAATTCTTGTCTTTCTTATATATATATGGAGGAGTGCGTTTGAGATACGCC**A**GGACTCTAGAGAATTC
TTCTCTTTCATTTATCTACGATAAAGTCTTCGTTTCTCCTCTCATTCTTTCACTAGAATAGAGAAAAAGAAGGACAATTG
CAAAAGCTCTTTTTATAATTCAATCTTCCTTCTCAATTTTTTTTTCTATTTTCAAGCAAACCAACGGATTTTGACGTTCG
TCTTTTCGTTATCTTTCAAAGTTAGCTATTTTAAAGAAAAAATGGATTTAAACTTATGTTTGCTATGTGGTAACTCCATC
GATGCCGAAGGATTGTATTGCTCAAATGAATGTCGAATTCAGGA

tna1 carboxylic acid transmembrane transporter

TAAATTTGTCAGTTTTGTGT**TCGCAGTTTAAA**AAGCTACGTATAATAAACGAATAAAAGGCTTTACAATGATCTAATCAA
TTTCAGCCTT**TATATTTACCGA**GGCATAAGTTGTCCAATCATATTATTTTTTAGAACTAATATTCAATCATTGCAAGCTA
TTTCCAGATATCCGGATGCATGCTCAACCGATCAGCCGGGTTATATATTGACTGTACATGGCTCGACGAAAAAT**G**CTC**A**C
TGCTTTGAGATCGGTC**A**TC**A**ACATCCATATATTCATCTTTTTTTTCTTCTCCTTAATAGCTTGGGGAATTGTATTTCCTT
TCAGATTTTGGAATTTCGCGGTACCTTTTCTAATACTGGTTTTCACTAATTCACCTTCACTTAAGCGAAAAACCCAAAAC
TTAATATCGCTGCGAAAATCATTTGTATCAGATTGACATTAACCATTCGATTGATCCCTAGTAGTTTCACTTATATCCTA
CTAATTCCCTAATTATATTGCATTTAATTTACCTTTTATAAAAAGACAACTTACATACGAAAAAGTTTTAAGGTCGTCGT
TTTTCGTTTTCAAAAAAAAAAAAAAATATGAAGTCTTTAAGTCACACTAGCAGTAATAAGAGCAATGGCAGCATCTTTACC
AAAGCGGATGAAAGTGAAAAAGTCATCAGCCGTTCTAACACTGCTAGTCCAAT

The list of 16 Pho7-dependent phosphate starvation-induced genes (see Fig. 4) includes those, other than *pho1*, *tgp1*, and *pho84*, for which transcription start sites have been mapped (13). A start site and 5'-UTR have not been annotated for SPAC26F1.11.

Figure S3: <u>Coregulation of *ecl3* with neighboring genes *pho1* and *pho84*</u>.

strain	log2 fold RNA change		
	ecl3	***pho1***	***pho84***
asp1-H397A	+2.75	+2.56	+2.12
asp1-(386stop)	+3.69	+3.40	+2.22
asp1-(493stop)	+3.71	+3.63	+2.43
aps1Δ	+2.03	+1.71	+1.45
rpb1-CTD-(S5•S5A)	+2.82	+2.70	+1.22
rpb1-CTD-(P6•P6A)	+2.83	+2.82	+1.24
duf89Δ	+2.06	+1.73	+1.30
rad24Δ	+3.44	+3.38	–
seb1-G476S	+1.95	+1.96	–
asp1-D333A	−2.21	−4.37	−3.83
pin1Δ	−1.56	−2.74	−2.42
ssu72-C13S	−1.61	−2.47	−2.24
ppn1Δ	−1.62	−3.83	−3.81
swd22Δ	−1.63	−3.57	−3.43
kcs1-L338R	−2.17	−1.77	−1.43
kcs1-E834K	−2.02	−1.95	−1.47
kcs1-R332T	−3.52	−6.00	−4.92

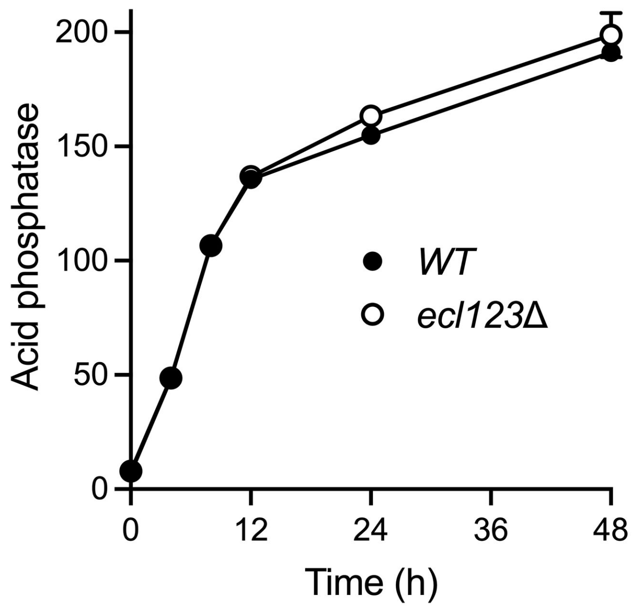

Figure S4. Starvation-induced Pho1 acid phosphatase expression is unaffected by pan-*ecl* deletion. Mid-log cultures of wild-type or *ecl123*Δ cells grown in YES medium were harvested, washed with water, transferred to ePMGT medium with no phosphate, and incubated for 48 h at 30˚C. Cells harvested prior to (0 h) and at sequential times after transfer to phosphate-free medium were washed with water and resuspended in water. To quantify acid phosphatase activity, reaction mixtures (200 µl) containing 100 mM sodium acetate (pH 4.2), 10 mM *p*-nitrophenylphosphate, and cells (ranging from 0.01 to 0.1 A_{600} units) were incubated for 5 min at 30°C. The reactions were quenched by addition of 1 ml of 1 M sodium carbonate, the cells were removed by centrifugation, and the absorbance of the supernatant at 410 nm was measured. Acid phosphatase activity is expressed as the ratio of A_{410} (*p*-nitrophenol production) to A_{600} (cells). The data shown are averages (±SD) of three assays using cells from three independent cultures.

Table S6. Fission yeast strains used in this study.

Strain	Genotype	Source
AS478	*h- Sp1 leu1-32 ura4-D18 his3-D1 ade6-m216*	Sanchez et al. 2019 (24)
AS479	*h+ Sp2 leu1-32 ura4-D18 his3-D1 ade6-m210*	Sanchez et al. 2019 (24)
AGP83	*h- pho7Δ::natMX*	Schwer et al. 2017 (4)
ASY96	*h- ecl3Δ::kanMX*	This study
ASY118	*h+ ecl2Δ::natMX*	This study
ASY121	*h+ ecl1Δ::hygMX*	This study
ASY128	*h- ecl1Δ::hygMX ecl3Δ::kanMX*	This study
ASY136	*h- ecl1Δ::hygMX ecl2Δ::natMX ecl3Δ::kanMX*	This study
ASY124	*h- atg1Δ::hygMX*	This study

All strains are *leu1-32 ura4-D18 his3-D1* and either *ade6-m216 or ade6-m210*.
